# Low Female Gametophyte Fertility Contributes to the Low Seed Formation of the Diploid Loquat [*Eriobotrya Japonica (Thunb.)* Lindl.] Line H30-6

**DOI:** 10.3389/fpls.2022.882965

**Published:** 2022-05-23

**Authors:** Qingqing Xia, Jiangbo Dang, Peng Wang, Senlin Liang, Xu Wei, Xiaolin Li, Suqiong Xiang, Haiyan Sun, Di Wu, Danlong Jing, Shumin Wang, Yan Xia, Qiao He, Qigao Guo, Guolu Liang

**Affiliations:** ^1^Key Laboratory of Horticulture Science for Southern Mountains Regions of Ministry of Education, College of Horticulture and Landscape Architecture, Southwest University, Chongqing, China; ^2^State Cultivation Base of Crop Stress Biology for Southern Mountainous Land of Southwest University, Academy of Agricultural Sciences, Southwest University, Chongqing, China; ^3^Economic Crops of Ziyang City, Ziyang City, China; ^4^America Citrus Research and Education Center, University of Florida, Gainesville, FL, United States

**Keywords:** seedless fruit, male sterility, female sterility, gametophyte differentiation and development, meiosis

## Abstract

Loquat is a widely grown subtropic fruit because of its unique ripening season, nutrient content, and smooth texture of its fruits. However, loquat is not well-received because the fruits contain many large seeds. Therefore, the development of seedless or few-seed loquat varieties is the main objective of loquat breeding. Polyploidization is an effective approach for few-seed loquat breeding, but the resource is rare. The few-seed loquat line H30-6 was derived from a seedy variety. Additionally, H30-6 was systematically studied for its fruit characteristics, gamete fertility, pollen mother cell (PMC) meiosis, stigma receptivity, *in situ* pollen germination, fruit set, and karyotype. The results were as follows. (1) H30-6 produced only 1.54 seeds per fruit and the fruit edible rate was 70.77%. The fruit setting rate was 14.44% under open pollination, and the other qualities were equivalent to those of two other seedy varieties. (2) The *in vitro* pollen germination rate was only 4.04 and 77.46% of the H30-6 embryo sacs were abnormal. Stigma receptivity and self-compatibility in H30-6 were verified by *in situ* pollen germination and artificial pollination. Furthermore, the seed numbers in the fruits of H30-6 did not significantly differ among any of the pollination treatments (from 1.59 ±0.14 to 2 ± 0.17). (3) The chromosome configuration at meiotic diakinesis of H30-6 was 6.87I + 9.99II + 1.07III +0.69IV +0.24V (H30-6), and a total of 89.55% of H30-6 PMCs presented univalent chromosomes. Furthermore, chromosome lagging was the main abnormal phenomenon. Karyotype analysis showed that chromosomes of H30-6 had no recognizable karyotype abnormalities leading to unusual synapsis on the large scale above. (4) The abnormal embryo sacs of H30-6 could be divided into three main types: those remaining in the tetrad stage (13.38%), those remaining in the binucleate embryo sac stage (1.41%), and those without embryo sacs (52.82%). Therefore, we conclude that the loquat line H30-6 is a potential few-seed loquat resource. The diploid loquat line H30-6 was with low gametophyte fertility, which may be driven by abnormal meiotic synapses. The low female gamete fertility was the main reason for the few seeds. This diploid loquat line provides a new possibility for breeding a few-seed loquat at the diploid level.

## Introduction

Loquat (*Eriobotrya japonica*), which originated in China, is a globally grown subtropical fruit and its fruits have high nutrition and a delicious taste, consumed directly or made into juices, jams, wines, and syrups (Lin et al., 1999; Lin, 2008). There are large seeds in loquat fruits, which is inconvenient for eating and processing. Therefore, the development of seedless and few-seed varieties has been the main goal of loquat breeders.

For a long period of time, polyploidization has been an effective approach to breeding seedless and few-seed loquat varieties. Early in 1984, Huang et al. (1984) reported that a tetraploid line (‘Min No. 3') was obtained *via* colchicine induction, and the majority of fruits (91.3%) had only one seed. In the 1990s, triploids were employed to produce seedless loquats by Liang, and seedless varieties including “Wuhe Guoyu” (Guo et al., 2016), “Huajinwuhe No. 1” (Dang et al., 2019a), and “Huayu Wuhe No. 1” (Dang et al., 2019b) have since been released. However, hormone treatment is necessary to increase the fruit setting rate of triploid loquat varieties (Maria et al., 2013). Some triploid lines were found to produce few-seed fruits when pollinated by diploid lines without hormone treatment (Kikuchi et al., 2014; Yang, 2021). In recent years, some tetraploids have been applied to breeding triploids on a large scale (Liang et al., 2018; Wang et al., 2021).

At the diploid level, seedless fruits could be obtained *via* hormone regulation, but the fruit qualities have been proven to be significantly lower than those of the seeded fruits (Gu and Zhang, 1990; Zhang et al., 1999; Chen et al., 2006; Deng, 2009b; Maria et al., 2013). “Tai Cheng No. 4” was reported to be a loquat variety with 1.3 seeds per fruit, and fruits with one seed were observed for up to 72.6% (Huang and Xu, 1980). However, there is no further information about this variety. An investigation reported by Jiang et al. (2009) indicated that 15 out of 128 lines bear fruits with 1–2 seeds, and there are two of the lowest lines (Guangmian and Hongxing), with 1.2 seeds per fruit. Zhang et al. (2014) reported that there were only 1.12 seeds per fruit of “Chuannong No.1”; however, the fruit setting rate of this variety was very low. Therefore, it can be concluded based on the above information that few-seed loquat resources are scarce.

It is logical that the abnormal meiosis of polyploids contributes to low fertility gametes, thus leading to seedless or few-seed fruit (Maria et al., 2013; Xu et al., 2016). But the few-seed mechanism of diploid loquat may be a more complex issue. While there have only been a few reports concerning this issue. Zhang et al. (2014, 2015) reported that gametophytic self-incompatibility resulted in the few-seed of “Chuannong No. 1”, and pollen inactivation caused by high temperatures contributed to a low fruit setting rate and few-seed fruit of “Chuanzao”. Mutant shoot breeding, chance seedling selection, and cross-breeding play an important role in the seedless variety breeding (Ledbetter and Ramming, 1989; Deng et al., 1996; Fan et al., 2004; Xu et al., 2006; Ye et al., 2006; Dong et al., 2013; Li and Wang, 2019; Li et al., 2019). So, it is important to discover more few-seed germplasm and identify the mechanism underlying the production of low numbers of seeds for breeding few-seed loquats.

An open-pollinated progeny of a white-fleshed variety “Huabai No.1”, named H30-6, was shown to bear fruits with few seeds. This line thus is an ideal material to breed new few-seed varieties and to research the mechanism underlying low seed formation. In the current study, we observed the stability of the H30-6 fruit set and quality traits over successive years. Moreover, the flower structure, gamete fertility, pollen mother cell (PMC) meiosis, stigma receptivity, *in situ* pollen germination, fruit setting, karyotypes, and so forth were studied to determine the factors underlying the low seed formation of loquat line H30-6. Taken together, the results can be referred to in few-seed loquat variety breeding at the diploid level.

## Materials and Methods

### Plant Materials

From 2016 to 2021, we obtained materials from vigorously growing plants in the orchard of fruit germplasm resources belonging to the Key Fruit Lab, of the College of Horticulture and Landscape Architecture, Southwest University. H30-6 is an open-pollinated progeny of a white-fleshed variety “Huabai No.1”, which is red-fleshed. In the orchard, the H30-6 line was represented by one 10-year-old individual and five 3-year-old grafted trees. So, in this study, in addition to the H30-6 line (red-fleshed), we chose two varieties differing in fertility, namely, “Huabai No. 1” (white-fleshed, named H411) and “Jinhua No. 1” (red-fleshed, named B336), both of which were represented by 11-year-old grafted trees.

### Methods

#### The Yield and Fruit Quality

The yield of the 10-year-old tree of H30-6 was measured from 2019 to 2021, and the yield of the 3-year-old grafted trees was measured in 2021. In 2019, 70 naturally set H30-6 fruits were used to calculate the seed number per loquat fruit, which was repeated three times. From 2019 to 2021, fruits of the three varieties were used each year to assess fruit quality, including average fruit weight, seed number per fruit, fruit edible rate, fruit shape index (longitudinal diameter/transverse diameter of the fruit), soluble solids (measured using a sugar refractometer), total sugar (measured using Fehling's reagent titration), reducing sugar (measured using Fehling's reagent titration), titratable acid (measured using sodium hydroxide titration), sugar-acid ratio (total sugar/titratable acid), and vitamin C (measured using 2,6-dichlorophenol sodium indophenol). The fruit edible rate, fruit shape index, and sugar-acid ratio were calculated using the following formulas:


$$\begin{array}{l} \text{Fruit edible rate }\left(\%\right)\text{ = }\frac{\text{Flesh weight}}{\text{Fruit weight}}\;\times \text{ 100}\%\\ \text{Fruit shape index = }\frac{\text{Longitudinal diameter per fruit}}{\text{Transverse diameter per fruit}}\\ \text{Sugar acid ratio }\left(\%\right)\text{ = }\frac{\text{Total sugar}}{\text{Titratable acid}}\;\times \text{ 100}\% \end{array}$$

#### Flower Organ Observations and Quantification

The flower buds were dissected 1 day before opening (DBO) to count the flower organs and measure the stigma length. The evenness of stigma length was calculated as the ratio of the number of flowers with stigmas of the same length to the number of all flowers observed. There were 30 flowers of H30-6, H411, and B336 which were observed separately. Many of the anthers of the flowers dissected with 1 DBO were collected, dried naturally at 25°C, and stored at 4°C for subsequent experiments. We collected images of blooming flowers to show the anther dehiscence state of the three varieties in the field. Additionally, the dried anthers were observed and imaged under a Carl Zeiss Jena Axio Zoom version 16 microscope (Jena, Germany).

#### Pollen Morphology, Pollen Viability, and Pollen Quantity

To study pollen morphology, dried pollen grains were adhered onto the sample stage with conductive adhesive, coated with gold in a KYKY SBC-12 vacuum evaporation instrument, and observed under a Phenom ProX scanning electron microscope (Thermo Fisher Scientific, Waltham, MA, USA). For H30-6, H411, and B336, three pollen group photos were taken separately by scanning electron microscope to count the pollen with normal or abnormal shapes. The total numbers of pollen counted were 255, 159, and 297 for H30-6, H411, and B336, respectively.

There were 10 dried anthers which were placed into a 1.5 ml centrifuge tube and then crushed in 200 μl BK medium with tweezers to count the pollen in a blood counting chamber. The test was processed three times for H30-6, H411, and B336 separately.

We slightly modified Hu Shiyi's pollen viability method (Hu, 1993) to test pollen viability. The dry anthers were placed into a 1.5 ml centrifuge tube with 200 μl BK medium, and the tube was shaken to allow pollen dispersion until the pollen density was the same among H30-6, H411, and B336. Then, the anthers were removed with tweezers. Later at 45 min, 50 μl pollen suspension was transferred into a new centrifuge tube and then placed into the dark constant temperature incubator upside down at 25°C for 4 h. After culture and thorough shaking, 15 μl suspension was dropped onto a glass slide, covered with a coverslip, and observed and imaged using an Olympus (BX35) fluorescence microscope (Tokyo, Japan). At least 6 times were repeated in each material. Each repeat took at least three pictures of different visions. The germinated pollen and ungerminated pollen were counted in each repeat. The total numbers of the pollen count in the viability test were 2,743, 1,724, and 2,568 for H30-6, H411, and B336, respectively.

#### Anthers Development

Buds with anthers with microspore mother cell before meiosis or at meiosis stage and buds 1 DBO were collected, fixed in a 38% formalin-acetic acid-70% alcohol solution (FAA, volume ratio = 1:1:18) for 24 h, and then preserved in 70% alcohol solution at 4°C. Anthers were dissected from fixed buds for serial paraffin sections following the protocol described by Li (1987), including safranine staining, dehydration, transparency, wax immersion, and embedding. Thin serial sections of 8–10 μm were cut with a microtome (RM2235, Leica, Wetzlar, Germany). This was followed by safranine staining, dehydration, Fast Green counterstaining, and rehydration prior to examination under a light microscope (BX53, Olympus, Tokyo, Japan).

#### PMCs Meiosis

Anthers were dissected from buds, fixed in methyl alcohol: acetic acid solution (3:1) for 24 h, rinsed with deionized water, and then treated with 3% (w/v) cellulose, 1% (w/v) macerozyme pectinase, and 1% (w/v) snailase for 4 h. Slides were prepared using the chromosomal smear technique (Liang and Li, 1991) with Na_2_HPO_4_ solution prestaining for 30–40 min and 5% Giemsa staining for 10–15 min and then observed under a light microscope (BX53, Olympus, Tokyo, Japan).

#### Stigmas Receptivity

The benzidine staining method (Dafni et al., 2005) was used to assay stigma receptivity. The staining solution contained 1% benzidine, 3% H_2_O_2_, and H_2_O (volumetric ratio = 4:11:22), and 3% H_2_O_2_ was prepared when needed. Five flowers at 1 DBO of H30-6, H411, and B336 were chosen separately, and the stigmas were revealed by removing the sepals, petals, and anthers. The stained stigmas were observed and imaged under a Carl Zeiss Jena Axio Zoom version 16 microscope (Jena, Germany).

#### In Situ Pollen Germination

The aniline blue staining protocol was used to test *in vivo* pollen tube growth according to Hu (1982), and the tissues were observed under a light microscope (BX53, Olympus, Tokyo, Japan). Five pollination methods were used on H30-6 to test for *in situ* pollen germination. First, natural pollination with bags was applied: some branches with many flowers were bagged before the flowers opened, and then each flower was marked on the day it started opening. More than 20 flowers were collected and fixed 2, 3, and 4 days after opening (DAO). Second, open pollination was allowed to proceed: when the flowers naturally opened, they were marked. More than 20 flowers were collected and fixed 2, 3, and 4 DAO. Third, artificial selfing was performed: some branches with many flowers were bagged before the flowers opened, and the flowers 1 DBO were emasculated and pollinated with dry pollen of H30-6. More than 20 flowers were collected 2 days after pollination (DAP) and fixed. Fourth, cross-pollination with H411 was applied: some branches with many flowers were bagged before the flowers opened, and 1 DBO, the flowers were emasculated and pollinated with dry pollen of H411. More than 20 flowers were collected 2 DAP and fixed. Fifth, cross-pollination with B336 was conducted: several branches with many flowers were bagged before the flowers opened, and flowers at 1 DBO were emasculated and pollinated with dry pollen of B336. More than 20 flowers were collected 2 DAP and subsequently fixed.

#### Embryo Sac Observations

The ovules were separated from flowers 1 DBO, fixed in FAA for 48 h, and then transferred to 70% ethanol for 24 h. We followed the protocol of Zilli et al. (2015) for dehydration and removal, with the following modifications: 10% H_2_O_2_ (30 min), 10% H_2_O_2_ (2 h), 70% ethanol 2 h, 85% ethanol (2 h), 95% ethanol (2 h), 100% ethanol (2 h), 100% ethanol (overnight), methyl salicylate/ethanol 50:50 v/v (3 h), methyl salicylate/ethanol 75:25 v/v (3 h), methyl salicylate/ethanol 85:15 v/v (3 h), methyl salicylate (3 h), and methyl salicylate until observations performed through a light microscope (BX53, Olympus, Tokyo, Japan). The numbers of ovules observed in this test were 142, 289, and 123 for H30-6, H411, and B336, respectively.

#### Pollination Treatments

From 2017 to 2021, reciprocal crosses were performed among H30-6, H411, and B336, and pollen was collected and saved in the same way as germination in the *in situ* experiment. In the meantime, the flowers of H30-6, H411, and B336 were processed by natural pollination with bags, open pollination, and artificial selfing separately. Each year, the fruits in each treatment were harvested, and the numbers of fruits and seeds were counted. The fruit setting rate, average seed number per fruit, and seed ovule ratio were calculated using the following formulas:


$$\begin{array}{l} \text{Fruit setting rate}\left(\%\right)\\ \text{=}\frac{\text{Total fruit number of one treatment}}{\text{Total flower number of the treatment}}\times \text{100}\%\\ \text{Seed ovule ratio }\left(\%\right)\\ \text{=}\frac{\text{Total seed number of one treatment}}{\text{Flower number of the treatment}\times 10}\times \text{100}\%\\ \text{Seed number per fruit }(\%)\\ \text{=}\frac{\text{Total seed number of one treatment}}{\text{Total fruit number of the treatment}}\times \text{100}\% \end{array}$$

#### Chromosome Karyotype Analysis

Root tips and shoot tips were placed into 0.002 mol/L 8-hydroxyquinoline aqueous solution for 4 to 5 h in the dark, fixed in methyl alcohol: acetic acid solution (3:1, v/v) for 24 h, rinsed with deionized water, and then treated with 3% (w/v) cellulose and 1% (w/v) macerozyme for 3 h. Slides were prepared and observed as described for the meiosis slides. Chromosome karyotype analysis was according to Li and Chen (1985) standard. Chromosome types and relative lengths were determined according to Levan et al. (1964). While karyotype classification was performed according to the Stebbins (1971) standard, the asymmetrical karyotype coefficient was calculated as described by Arano (1963).

### Data and Image Processing

Microsoft Excel (Redmond, Washington, USA) and SPSS 17 software (IBM, Armonk, NY, USA) was applied for statistical analysis. Adobe Photoshop CS5 (Sam Jose, USA) was applied for image processing. The parameters in the fruit quality test, the pollen quantity, normal shape pollen ratio, germination pollen ratio, fruit setting rate, seed ovule ratio, and No. of seeds per fruit were presented by mean ± standard error (SE). ANOVA for mean comparisons (LSD/Dunnett T3, *P* <0.05) was performed. The percentage data were transformed using the arcsine square root transformation to normalization separately before the significant difference tests.

## Results

### The Yield of H30-6 and Fruit Quality of Naturally Seated Fruit Naturally Seated Fruit

**Figure 1:**
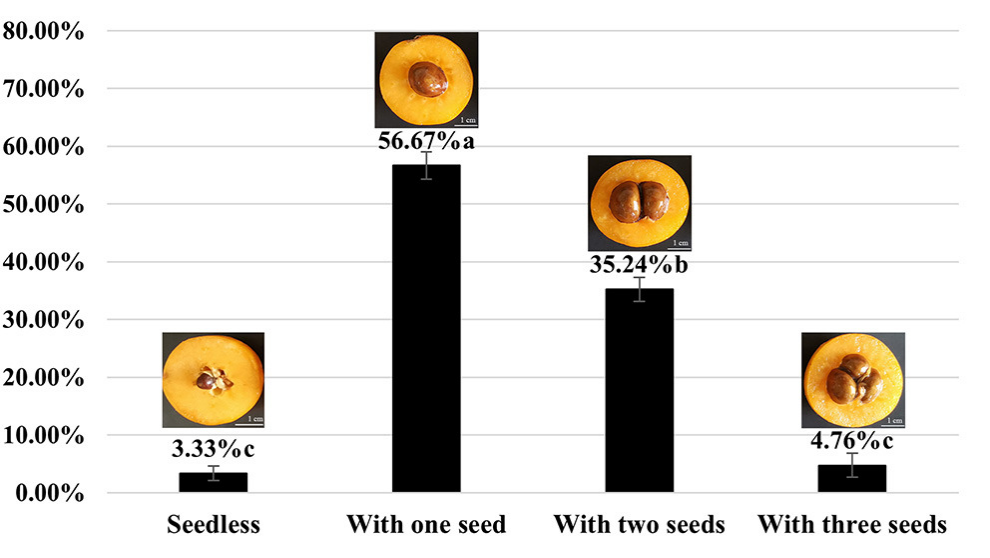
The proportion of H30-6 open-pollinated fruits with different seed numbers. The statistical differences in means of the ratio of fruits with different seed numbers were determined using the LSD test; different letters beside the bar indicate that the means are significantly different (*P* < 0.05); values are presented as mean (± SE).

The average yield of the 10-year-old tree of H30-6 was 14.43 kg (15.19, 18.36, and 9.75 kg, from 2019 to 2021 separately), and the average yield of the 3-year-old grafted tree was 7.3 kg. The results showed that the seed numbers of open-pollinated H30-6 fruits were between 0 and 3 (Figure 1), and the percentage of fruits with one seed was 56.67%, followed by two-seeded fruit (35.24%). Subsequently, similar observations were made in the last 2 years, when the average seed number per fruit was 1.54, which was significantly lower than that of the controls (H411, 4.63 ± 0.22; B336, 3.23 ± 0.12). All the results proved that loquat line H30-6 was a few-seed resource. Furthermore, the successive three-year fruit quality data revealed that H30-6 had the highest fruit edible rate (70.77 ± 0.61%), and the other detection indexes were equivalent to those of the two controls (Table 1). Therefore, loquat H30-6 can be used as a commercial variety.

**Table 1:** Fruit qualities during three consecutive years (2019 to 2021).

**Lines/varieties**	**H30-6**	**H411**	**B336**
Average fruit weight (g)	32.59 ± 1.47^b^	31.58 ± 0.86^b^	36.89 ± 0.77^a^
Seed number per fruit	1.54 ± 0.09^c^	4.63 ± 0.22^a^	3.23 ± 0.12^b^
Edible rate (%)	70.77 ± 0.61^a^	65.3 ± 1.03^b^	65.76 ± 0.69^b^
Fruit shape index	1.30 ± 0.02^a^	0.90 ± 0.01^c^	1.11 ± 0.02^b^
Soluble solids	9.94 ± 0.35^b^	12.43 ± 0.56^a^	9.28 ± 0.36^b^
Total sugar (g/100 ml)	7.61 ± 0.0.13^b^	10.72 ± 0.59^a^	7.66 ± 0.50^b^
Reducing sugar (g/100 ml)	6.75 ± 0.20^a^	6.93 ± 0.16^a^	5.80 ± 0.17^b^
Titratable acid (g/100 ml)	0.24 ± 0.01^b^	0.37 ± 0.03^a^	0.28 ± 0.01^a^
Sugar acid ratio	31.41 ± 0.60^a^	30.69 ± 1.29 ^a^	27.39 ± 1.16^b^
Vc (mg/100 ml)	4.65 ± 0.43^a^	3.82 ± 0.26^a^	3.98 ± 0.45^a^

*ANOVA for mean comparisons (LSD, P < 0.05) was performed among H30-6, H411, and B336 (the average fruit weight, fruit shape index, soluble solids, total sugar, and titratable acid were analyzed by Dunnett T3 test among different treatments). Lowercase letters indicate differences at the level of significance of 5%; values are presented as mean ± SE*.

### Flower Organs

**Figure 2:**
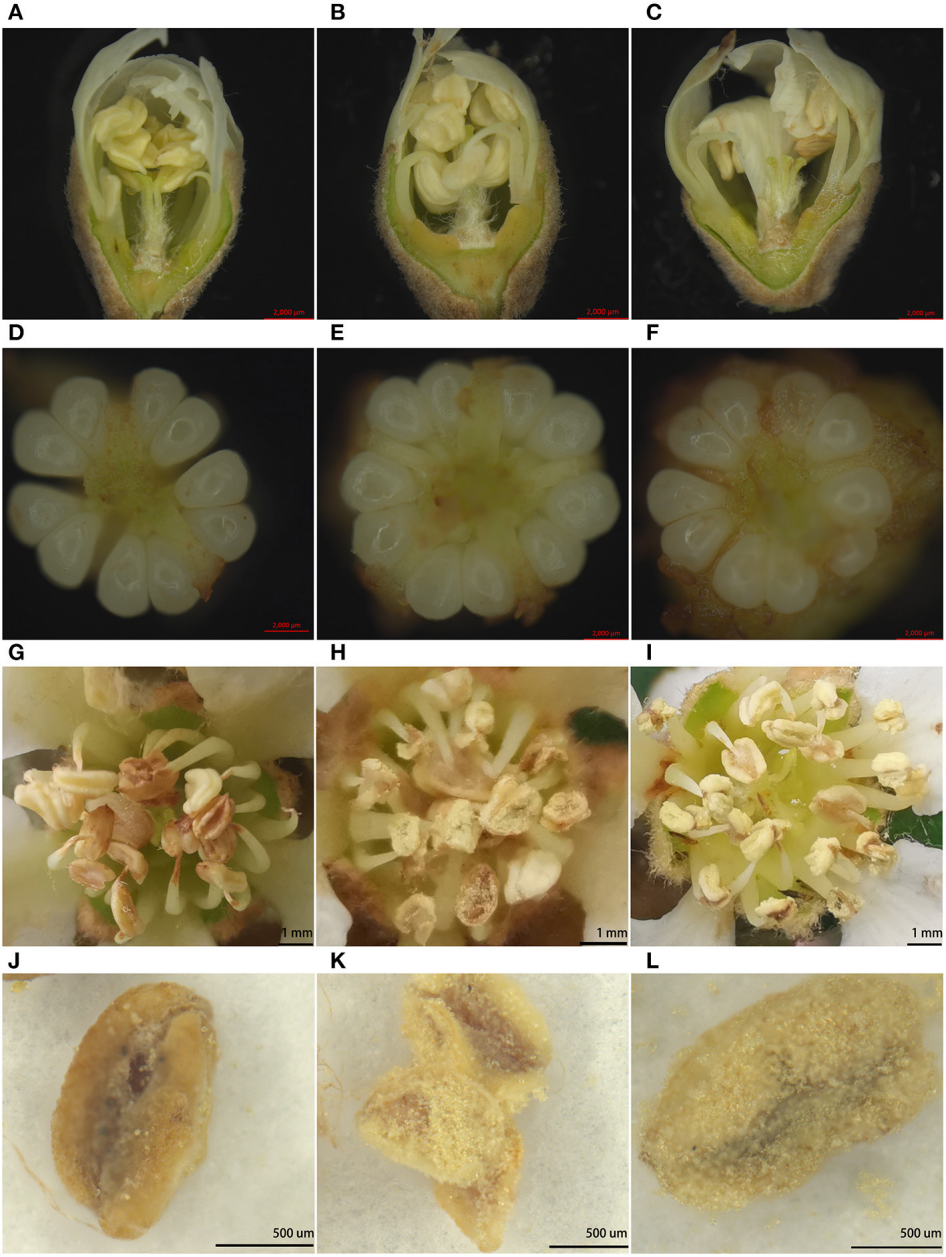
Images of flower organs: **(A–C)**, vertical sections of flower buds 1 day before opening [H30-6 **(A)**, H411 **(B)**, B336 **(C)**]; **(D–F)**, ovules in one ovary [H30-6 **(D)**, H411 **(E)**, B336 **(F)**]; **(G–I)**, field observations of anther dehiscence [H30-6 **(G)**, H411 **(H)**, B336 **(I)**]; **(J,K)**, dried anthers imaged under a Carl Zeiss Jena Axio Zoom. v16 microscope [H30-6 **(J)**, H411 **(K)**, B336 **(L)**].

Among the flower organs, we observed the numbers of petals, sepals, anthers, and ovules, and the stigma length evenness (5, 5, 20, 10, 80%) was equal to that of B336, but H411 had more anthers (21.87) and lower stigma length evenness (40%) (Table 2, Figures 2A–F). Therefore, the number of flower organs is not the reason for the low seed formation of H30-6. Field observations of anther dehiscence showed that H411 and B336 released large amounts of pollen while almost no pollen was released from H30-6 anthers (Figures 2G–I). Similarly, the dried anthers released little pollen. These results revealed that there are abnormalities in the anthers or pollen of H30-6 (Figures 2J–L).

**Table 2:** Flower organ numbers and evenness degree of stigma length of H30-6, H411, and B336.

**Lines/ varieties**	**Average petal number**	**Average sepal number**	**Average anther number**	**Average ovule numbers**	**Average style number**	**the evenness degree of stigma length (%)**
H30-6	5	5	20	10	5	80
H411	5	5	21.87	10	5	40
B336	5	5	20	10	5	80

### Pollen Morphology, Viability, and Quantity

**Figure 3:**
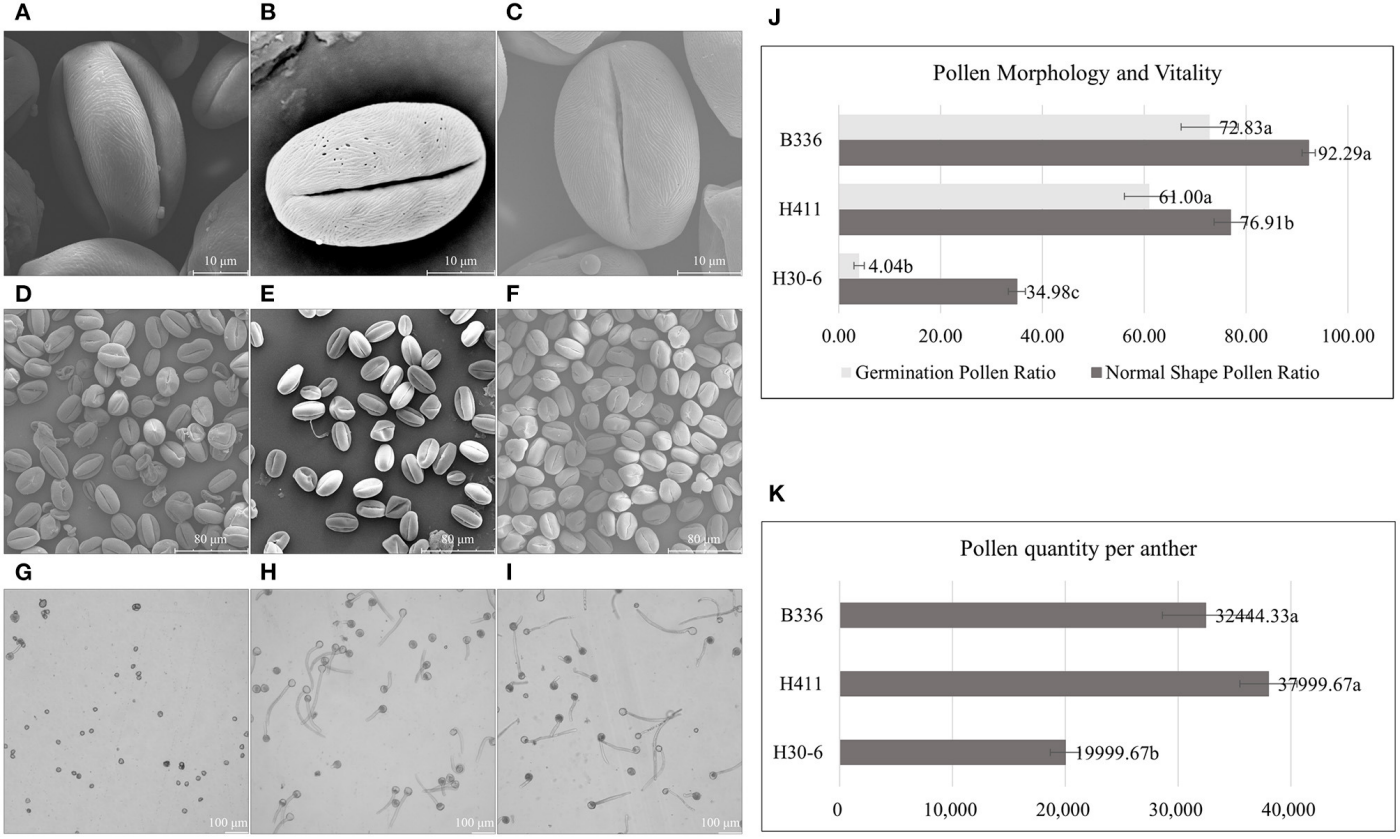
Pollen morphology as observed *via* scanning electron microscopy, images of pollen germination, numbers of normally shaped pollen grains and germinated pollen grains, and pollen quantity per anther: **(A–C)**, equatorial views of pollen grains [H30-6 **(A)**, H411 **(B)**, B336 **(C)**] showing the sexine with striate ornamentation and sparse holes between the ridges. **(D–F)**, pollen grains of H30-6, H411, and B336 under the scanning electron microscope. **(G–I)**, *in vitro* pollen germination of H30-6, H411, and B336, respectively. **(J)** normal shape pollen ratio and germination pollen ratio of H30-6, H411, and B336. **(K)** pollen quantity per anther. The statistical differences in means of normal shape pollen ratio, germination pollen ratio among H30-6, H411, and B336 were determined using the LSD test separately; different letters beside the bar indicate that the means are significantly different (*P* < 0.05); values are presented as mean (± SE).

The scanning electron microscopy results (Figures 3A–F) showed that the loquat pollen grains were medium in size, prolate, and tricolporate. We also found that the pollen sexine ornamentation of the three varieties was striated, with sparse holes between the ridges. However, there were significant differences in normal pollen ratios among H30-6, H411, and B336. The H30-6 line presented the lowest normal pollen rate (34.98%), while those of H411 and B336 were 76.91 and 92.29%, respectively (Figures 3D–F,J). The viability test presented similar results, as the germination rates of the H30-6, H411, and B336 lines were 4.04, 61, and 72.83%, respectively (Figures 3G–J). Thus, the H30-6 line exhibited low male fertility. According to Deng (2009a) standard, the H30-6 strain was a male-sterile resource, and the controls were both fertile. We also found that the pollen quantity of H30-6 was significantly lower than that of the two controls (Figure 3K).

### Anther Anatomical Structure

**Figure 4:**
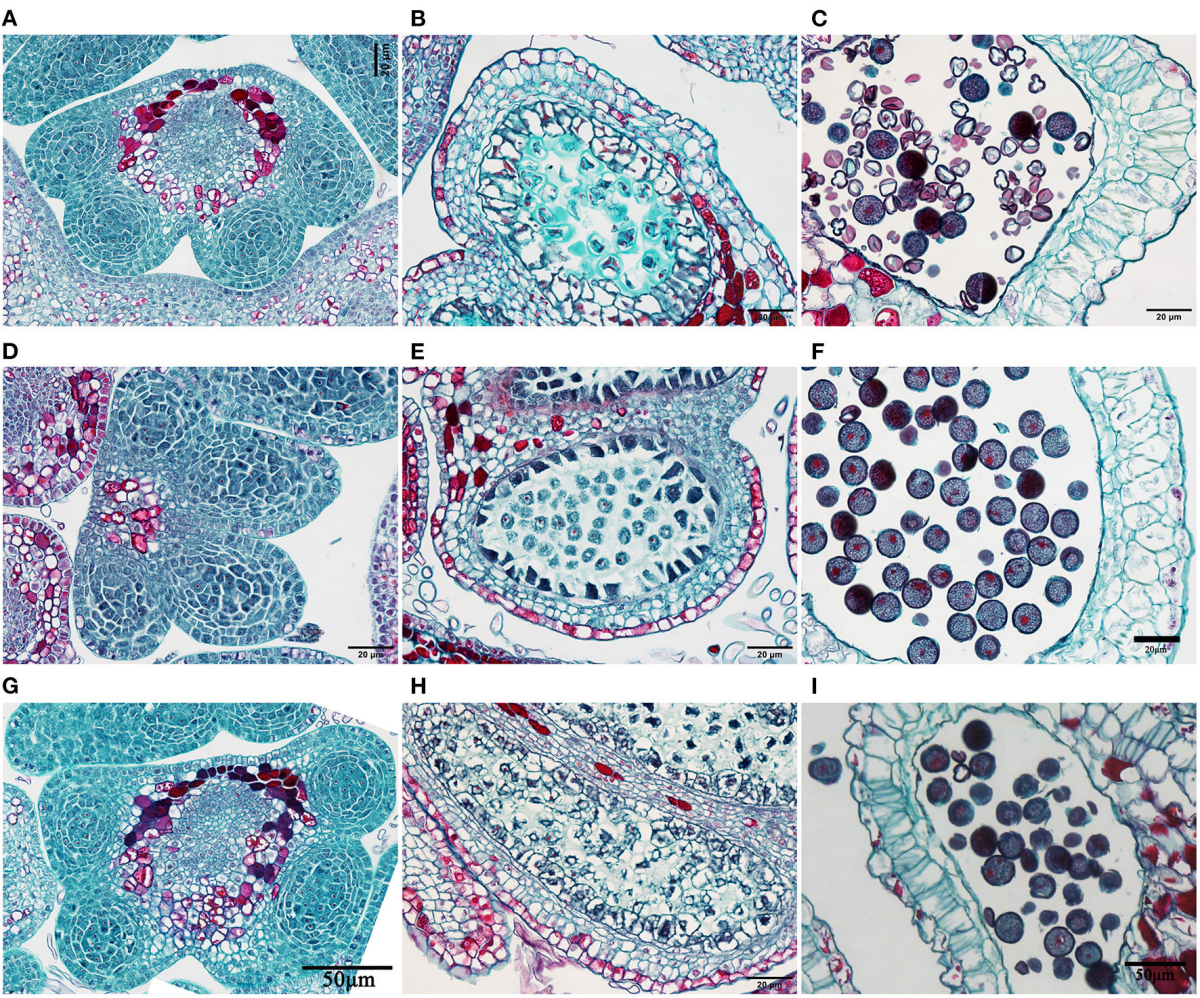
Images of anther slices: **(A–C)**, Anther of H30-6 at the microspore mother cell stage (with microspore mother cells before meiosis), tetrad stage of meiosis stage, as well as the mature pollen stage; **(D–F)** anther of H411 at the microspore mother cell stage, meiosis stage, and mature pollen stage; **(G–I)** anther of B336 at the microspore mother cell stage, tetrad stage, and mature pollen stage.

The anther paraffin sections revealed no obvious structural differences among H30-6, H411, and B336 during the developmental process (Figure 4). However, in male gametangia dissected from flower buds 1 DBO, the controls had plump pollen (Figures 4F,I), while the pollen of H30-6 was distorted and shrunken (Figure 4C). Therefore, we hypothesize that the reason for the lower pollen release involved the pollen mother cells, not the other tissues.

### PMCs Meiosis

**Figure 5:**
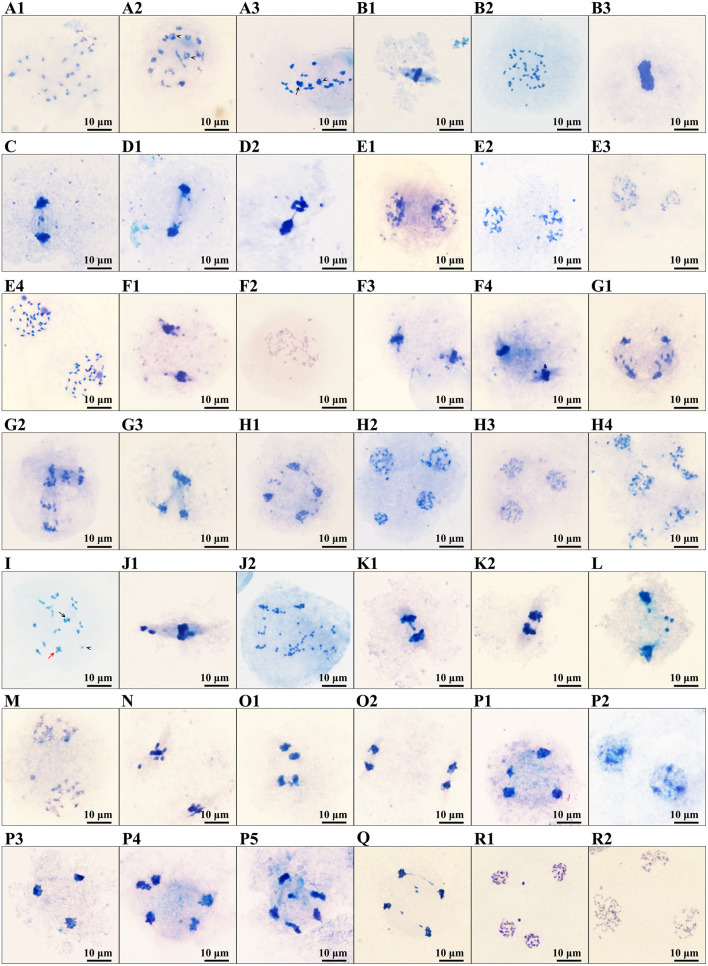
Images of abnormal phenomena of PMCs meiosis: **(A1–H4)**, images of PMCs meiosis of H30-6: **(A1–A3)**, diakinesis, **(A1)** showing the univalent, **(A2)** showing the trivalent, **(A3)** showing the quadrivalent (arrowhead) and pentavalent (arrow); **(B1–B3)**, metaphase I, **(B1)** showing a chromosomes lag, **(B2,B3)** showing doubled chromosomes; **(C)** showing chromosomes lag and micronucleus in anaphase I; **(D1,D2)**, Telophase I, showing chromosomes lag, micronucleus and the bridge of chromosomes; **(E1–E4)**, prophase II, **(E1)** showing the dissociative chromosome, **(E2)** showing the micronucleus, **(E3)** showing the unequal separating of chromosomes, **(E4)** showing the doubled chromosomes; **(F1–F4)**, metaphase II, **(F1)** showing chromosomes lag and micronucleus, **(F2)** showing doubled chromosomes, **(F3)** showing perpendicular spindle, **(F4)** showing splayed spindle; **(G1–G3)** anaphase II, **(G1)** showing the splayed spindle chromosomes lag, **(G2)** showing perpendicular spindle and chromosomes lag, **(G3)** showing cross spindle and micronucleus; **(H1–H4)**, telophase II, **(H1)** showing chromosomes lag and micronucleus, **(H2)** showing unequal separation of chromosomes, **(H3)** showing triad and micronucleus, **(H4)** showing polyad. **(I–P5)**, images of PMCs meiosis of H411: **(I)** showing the univalent (arrowhead), trivalent (arrow), and quadrivalent (red arrow); **(J1,J2)**, metaphase I, **(J1)** showing chromosomes lag, **(J2)** showing doubled chromosomes. **(K1,K2)**, anaphase I, **(K1)** showing chromosome lag and chromosome bridge, **(K2)** showing chromosomes bridge; **(L)** showing chromosomes lag and micronucleus in telophase I; **(M)** showing micronucleus in prophase II; **(N)** showing chromosomes lag in metaphase II; **(O1,O2)**, anaphase II, **(O1)** showing chromosomes lag and chromosomes bridges, **(O2)** showing chromosome bridge; **(P1–P5)** telophase II, **(P1)** showing unequal separating of chromosomes, **(P2)** showing dyad, **(P3)** showing triad and micronucleus, **(P4,P5)** showing polyad and micronucleus. **(Q–R2)**, images of PMCs meiosis of B336: **(Q)** showing chromosome lad and chromosomes bridge in anaphase II; **(R1)** showing micronucleus, **(R2)** showing a triad. Scale: 10 μm.

The PMCs of B336 behaved quite normally throughout all the meiosis stages. Additionally, the normal behavior rate of H411 PMCs ranged from 78.31 to 95.64%, whereas those of H30-6 ranged from 0 to 41.03% (Table 3, Figure 5, Supplementary Figure 2). The high-frequency abnormalities of H30-6 included incorrect chromosome pairing, chromosome lag, abnormal spindle formation, unequal division, and micronucleus formation. To analyze homologous chromosome pairing, chromosome configurations at diakinesis were observed and quantified. The results showed that the average chromosome configurations were 6.87I + 9.99II + 1.07III + 0.69IV + 0.24V (H30-6), 0.70I + 16.50II + 0.02III + 0.06IV (H411), and.01I + 16.99II (B336) (Supplementary Table 1). From these configurations, we found that the chromosome configuration of H30-6, H411, and B336 was mainly bivalent, however, H30-6 also showed a high frequency of univalents, and 89.55% of the H30-6 PMCs presented as univalent (Supplementary Table 1). Due to the randomness of the bipolar spindle fibers, univalent travel to one pole will be delayed. In the later meiosis process, chromosome lag was the main phenomenon, which accounted for 65.31, 95.24, 48, 55.13, 95, and 46.32% of the observations at metaphase I, anaphase I, telophase I, metaphase II, anaphase II, and telophase II, respectively (Table 3, Figure 5). Usually, lagging chromosomes are distributed randomly, as well as losing or forming micronuclei. Some of the immature mini-microspores grew to the mature pollen stage. This was proven by the pollen images obtained by scanning electron microscopy, which showed many small pollen grains. Therefore, we believe that the high frequency of univalent chromosomes is the reason for the chromosome lag, which may cause a decrease or an increase in the inherited material, leading to an influence on the fertility of the pollen. In addition, we found that the percentages of PMCs with doubled chromosomes were as high as 16.33 and 41.03% at metaphase I and metaphase II, respectively.

**Table 3:** Counting of pollen mother cell (PMC) meiosis phenomena.

**Meiosis stage**	**Phenomenon**	**Lines/varieties**					
		**H30-6**		**H411**		**B336**	
		**Number of PMCs**	**Percentage (%)**	**Number of PMCs**	**Percentage (%)**	**Number of PMCs**	**Percentage (%)**
Metaphase I	Normal	10	20.41	490	81.53	135	97.82
	With chromosomes lag	32	65.31	104	17.3	3	2.17
	Chromosomes doubled	8	16.33	7	1.16		
	Total number of PMCs	49		601		138	
Anaphase I	Normal	0	0	277	90.52	112	100
	With chromosomes lag	20	95.24	24	7.84		
	With micronucleus	8	38.1				
	With chromosomes bridge			5	1.63		
	Total number of PMCs	21		306		112	
Telophase I	Normal	7	9.33	329	95.64	58	100
	With chromosomes lag	36	0.48	15	4.36		
	With chromosomes bridge	1	1.33				
	With micronucleus	32	42.67				
	Total number of PMCs	75		344		58	
Prophase II	Normal	15	31.91	255	93.41	219	100
	With dissociative chromosomes	17	36.17				
	With micronucleus	12	25.53	18	6.59		
	Unequal separation of chromosomes	2	4.26				
	Chromosomes doubled	1	2.13				
	Total number of PMCs	47		273		219	
Metaphase II	Normal	5	3.21	261	84.74	148	98.01
	With chromosomes lag	86	55.13	43	13.96	3	1.99
	Chromosomes doubled	64	41.03				
	With micronucleus	46	29.49	4	1.3		
	With perpendicular spindle	6	3.85				
	With splayed spindle	9	5.77				
	Total number of PMCs	156		308		151	
Anaphase II	Normal	0	0	195	78.31	86	98.85
	With chromosomes lag	57	91.94	15	6.02	1	1.15
	With micronucleus	21	33.87	9	3.61		
	With chromosomes bridge			30	12.05		
	With splayed spindle	8	12.9				
	With perpendicular spindle	2	3.23				
	With cross spindle	1	1.61				
	Total number of PMCs	62		249		87	
Telophase II	Normal	17	7.36	299	81.25	580	96.99
	With chromosomes lag	107	46.32			13	2.17
	Unequal separation of chromosomes	50	21.65	3	0.82		
	With micronucleus	97	41.99	38	10.33	2	0.33
	Dyad			2	0.54		
	Triad	11	4.76	14	3.8	3	0.5
	Polyad	5	2.16	12	3.26		
	Total number of PMCs	231		368		598	

### Stigma Receptivity and *in situ* Pollen Germination

**Figure 6:**
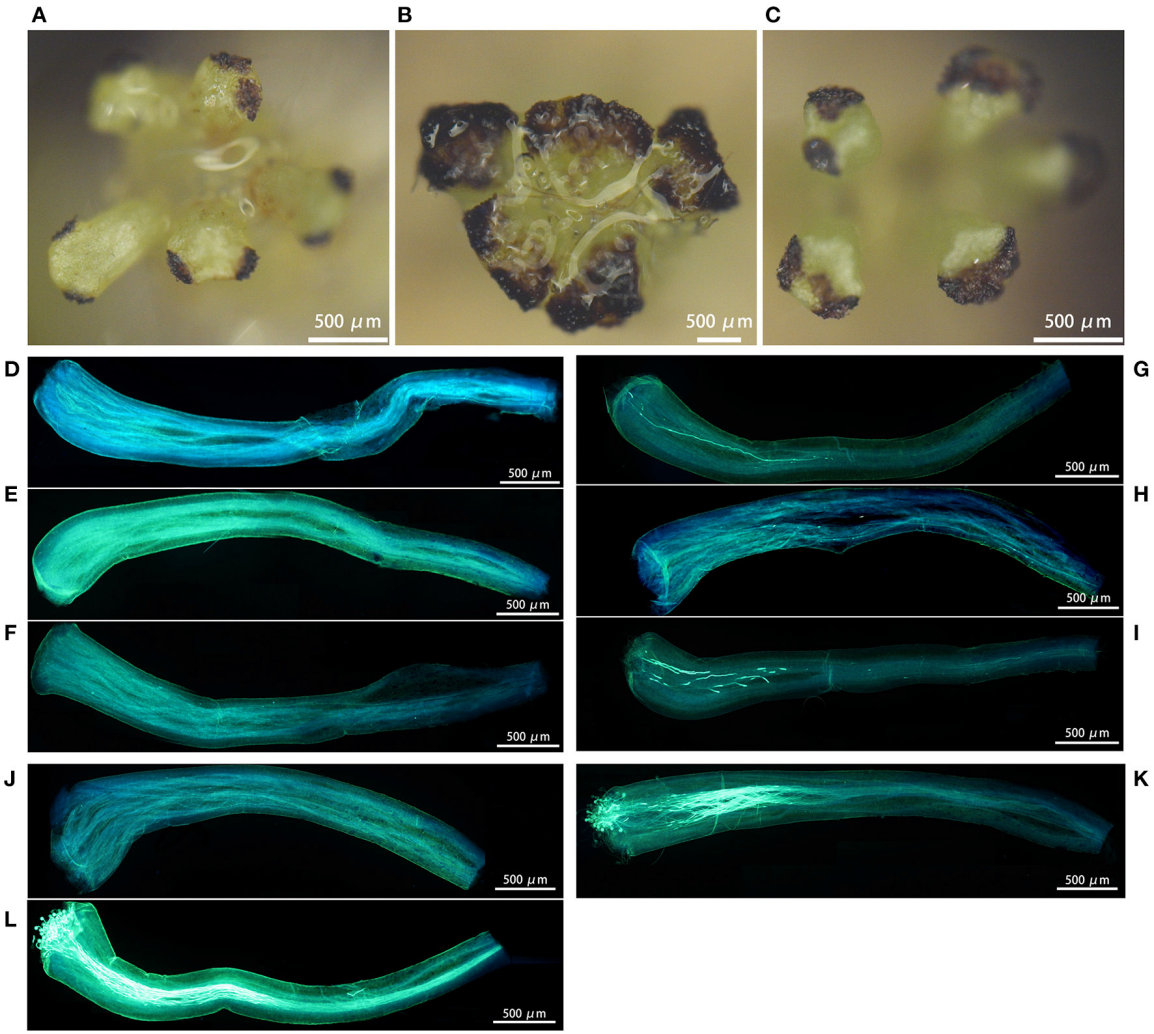
Images of the stigmas receptivity test and pollens *in situ* germination: **(A–C)** the stained area showing the stigmas receptivity of H30-6, H411, and B336 respectively. **(D–F)** showing pollen *in situ* germination at 2, 3, and 4 days after H30-6 bagging natural pollination receptivity. **(G–I)** showing pollen *in situ* germination at 2, 3, and 4 days after H30-6 open pollination receptivity. **(J–L)** showing pollen *in situ* germination at 2 days after H30-6 artificial selfing, pollination with pollens of H411, and pollination with pollens of B336.

We observed that loquat stigmas were heart-shaped or triangular. The stigmas of H30-6, H411 and B336 were stained dark brown (Figures 6A–C), meaning that all the stigmas were receptive. However, the stained areas showed some differences, especially those on the stigmas of H30-6, where only the small areas of two opposite angles were stained. The aniline blue staining results showed that the pollen germinated and that the pollen tubes passed through the styles, which also indicated that the stigma receptivity was normal. We calculated the percentages of stigmas with pollen, styles with germinated pollen, and styles penetrated by pollen tubes for each of the five pollination methods. For the first method (natural pollination with bag), there was no pollen on the stigmas and no pollen germination on the styles, regardless of whether the observation was made 2, 3, or 4 DAO (Figures 6D–F, Supplementary Table 2). For the second method (open pollination), from 2 DAO to 4 DAO, the percentages of stigmas with pollen, styles with germinated pollen, and styles with pollen tubes that passed through increased (21.7, 41.18, and 46.67%; 21.71, 39.5, and 45.56%; 0, 4.2, and 16.67%; respectively) (Figures 6G–I, Supplementary Table 2). The staining results of styles pollinated with H30-6 pollen (2 DAP) showed that the percentages of stigmas with pollen, styles with germinated pollen, and styles with pollen tubes that passed through were much lower than the percentages observed under pollination with H411 and B336 pollen (Figures 6J–L, Supplementary Table 2). These results suggest that H30-6 pollen can germinate on H30-6 styles but that its low pollen viability may cause a lower *in situ* germination rate and a lower percentage of style pollen tube passage, also proving that the natural fruits of H30-6 are likely hybrid fruits.

### Embryo Sac Structure

**Figure 7:**
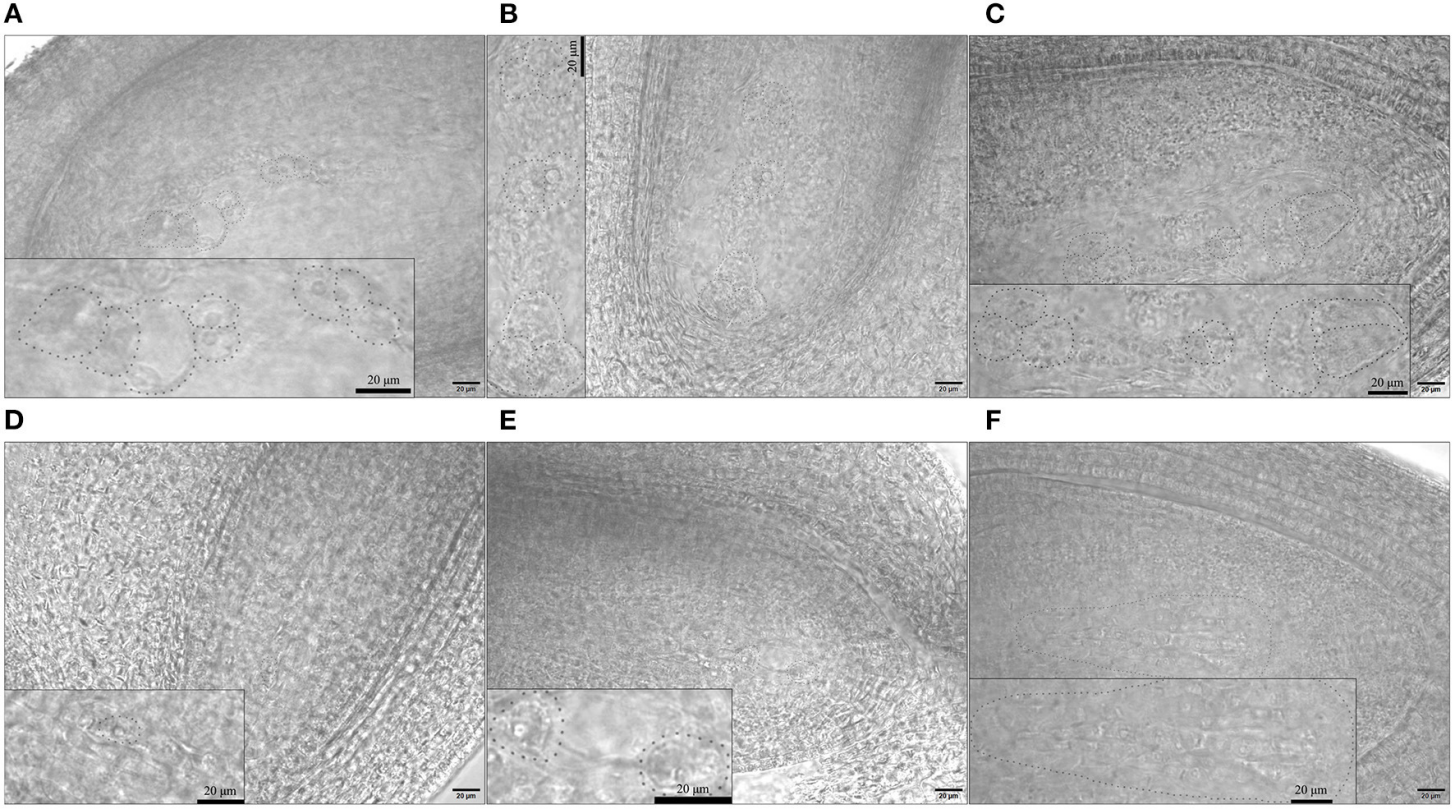
Images of normal and abnormal embryo sacs: **(A–C)** normal embryo sacs of H30-6, H411, and B336, respectively. **(D–F)** abnormal embryo sacs of H30-6: **(D)** embryo sac at the tetrad stage, **(E)** embryo sac at the binucleate embryo sac stage, **(F)** without embryo sac. The embryo sac area was magnified in the small box on each image. Scale: 20 μm.

As we observed ovules at 1 DBO, the embryo sacs should have been mature with 8 nuclei. H30-6, H411, and B336 all had normal embryo sacs, but the normal embryo sac rate of H30-6 was only 22.54%, which was obviously lower than that of the controls (H411 71.79%, B336 95.93%) (Figure 7, Supplementary Table 3). Each ovary had 10 ovules, so in H30-6, the normal ovule number per ovary was 2.25. This number was close to the average seed number of H30-6 natural fruits (1.54), so we speculate that embryo sac abnormality may be the most restrictive factor for lower seed formation. The abnormal embryo sacs could be divided into three main kinds: those remaining in the tetrad stage (with one functional macrospore or with four macrospores, 17.27%), those remaining in the binucleate embryo sac stage (1.82%), and those without an embryo sac (68.18%) (Figure 7, Supplementary Table 3). We hypothesize that majority of functional macrospores were disabled, which caused the differentiation shutoff or degradation of female gametes.

### Fruit and Seeds Obtained Through Various Pollination Treatments

The pollination treatments performed over consecutive years showed that when artificial selfing was applied to H30-6, the average fruit setting rate, average seed number per fruit and average seed ovule ratio were 9.93 ± 6.08%, 1.82 ± 0.28%, and 2.11 ± 1.44%, respectively (Table 4). Therefore, we believe that H30-6 is a self-compatible line. H30-6 pollination with H411 or B336 pollen dramatically improved the fruit setting rate (25.2 ± 6.23% and 26.68 ± 6.37%, respectively) compared with those under artificial selfing (9.93 ± 11.64% and 0.47 ± 2.94%) and natural pollination with bagging (2.13 ± 5.46% and 0.09 ± 1.24%) (Table 4). When H30-6 was open-pollinated, the fruit setting rate was significantly increased than those under natural pollination with bagging. These results showed the low fertility of H30-6 pollen was a limiting factor for the fruit setting. However, all pollination treatments to H30-6 showed no significant difference in seed number per fruit (from 1.59 ± 0.14 to 2 ± 0.17), which proved that the male gametes were not the main reason for the low seeds' formation in the natural fruits of H30-6 and may indicate that at least 1 to 2 seeds were usually needed for the loquat fruit set of H30-6 (Table 4). The average seed number per fruit was also close to the former results of the number of ovules with normal embryo sac (2.25) in the ovary of H30-6. All these results proved that embryo sac abnormality was the most important restrictive factor for seed formation of H30-6. The seed ovule ratios were dramatically increased when H30-6 was pollinated with H411 or B336 pollen than that natural pollinated with the bag. But there was no significant difference in seed ovule ratio among the four pollination treatments, pollination with pollen of H411, pollination with pollen of B336, open pollination, and artificial selfing when H30-6 was the female parent (Table 4). Meanwhile, the fruit setting rate and seed ovule ratio were without significant difference among pollination treatments (pollinated with pollen of B336 or H30-6, and artificial selfing) to H411 (Table 4). There was no significant difference in fruit setting rate and seed ovule ratio among pollination treatments (pollinated with pollen of H30-6 or H411, and artificial selfing) to B336 (Table 4). The results above showed that H30-6 can be used as a male parent in loquat breeding.

**Table 4:** The continuous years of pollination treatments results (2018 to 2021).

**Female parent (♀)**	**Pollination treatment**	**No. of flowers**	**No. of fruits**	**No. of seeds**	**Fruit setting rate (%)**	**No. of seeds pre fruit**	**Seed ovule ratio**
H30-6	×H411 (♂)	903	196	360	25.20 ± 6.23^a^	1.90 ± 0.15	4.64 ± 1.04^a^
	×B336 (♂)	904	204	400	26.68 ± 6.37^a^	2.00 ± 0.17	5.60 ± 1.73^a^
	Artificial selfing	887	96	200	9.93 ± 6.08^bc^	1.82 ± 028	2.11 ± 1.44^ab^
	Open pollination	720	159	261	14.20 ± 3.84^ab^	1.59 ± 0.14	2.28 ± 0.73^ab^
	Bagging natural pollination	1,203	6	11	0.47 ± 0.35^c^	1.88 ± 0.13	0.09 ± 0.06^b^
H411	×H30-6 (♂)	762	144	547	19.06 ± 8.94^ab^	3.22 ± 0.72	7.29 ± 4.70^ab^
	×B336 (♂)	780	222	874	28.22 ± 10.63^ab^	3.63 ± 0.40	11.08 ± 5.39^ab^
	Artificial selfing	943	287	1,187	31.56 ± 10.83^a^	3.89 ± 0.60	12.93 ± 5.79^a^
	Open pollination	585	98	346	13.15 ± 4.26^ab^	3.39 ± 0.49	4.90 ± 2.46^ab^
	Bagging natural pollination	1219	81	224	8.40 ± 3.73^b^	2.62 ± 0.33	2.41 ± 1.15^b^
B336	×H30-6 (♂)	731	167	574	20.68 ± 10.53^ab^	2.66 ± 0.70	6.96 ± 4.64^ab^
	×H411 (♂)	706	256	932	35.70 ± 7.45^ab^	3.62 ± 0.63	13.29 ± 4.03^ab^
	Artificial selfing	618	260	953	53.20 ± 20.23^a^	3.47 ± 0.41	18.71 ± 6.86^a^
	Open pollination	615	104	302	12.33 ± 1.77^b^	3.06 ± 0.52	3.55 ± 1.16^b^
	Bagging natural pollination	1,216	60	138	10.86 ± 7.95^b^	2.22 ± 0.15	2.55 ± 1.95^b^

*ANOVA for mean comparisons (LSD, P < 0.05) was performed among different treatments of the same female parent (the seed ovule ratio was analyzed by Dunnett T3 test among different treatments when H30-6 was a female parent). Lowercase letters indicate differences at the level of significance of 0.05; values are presented as mean ± SE*.

### Somatic Cell Karyotypes

The small amount and low vitality of pollen and the differentiation shutoff or degradation of the female gametes showed that H30-6 had low gametophyte fertility, which caused the low seed formation of the loquat fruit. We believe that there must be some common reasons for the low gametophyte fertility. As the abnormal meiosis of PMCs caused the male gametes to be sterile and the disabled functional macrogametes came from the MMCs, we observed the chromosomes of H30-6 to try to determine a common underlying factor.

**Figure 8:**
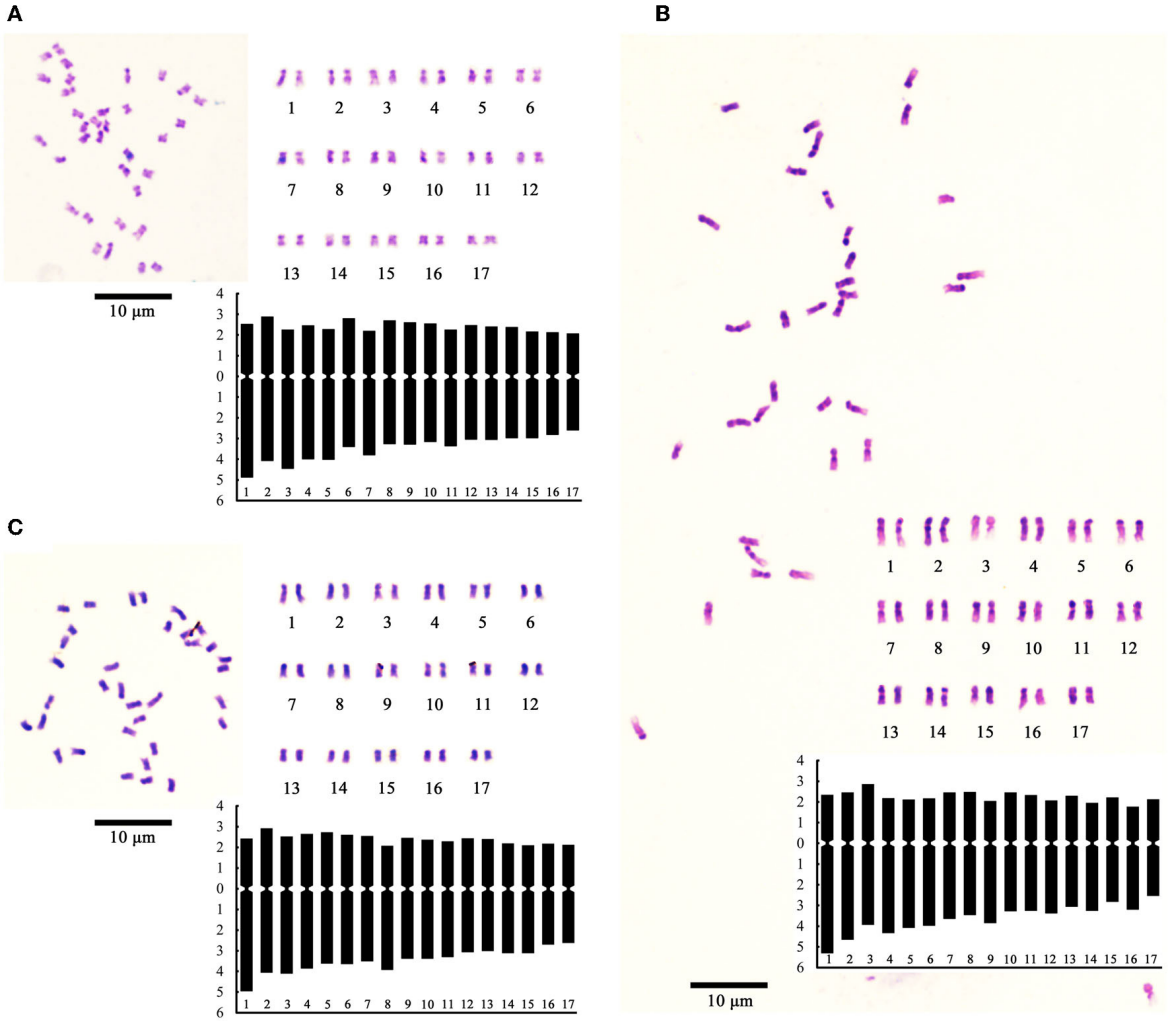
Images of chromosome photo, karyogram, and ideogram: **(A–C)** showing chromosome images, karyograms, and idiograms of H30-6, H411, and B336 respectively.

The chromosome number shoot and stem-tip cells showed that H30-6 was diploid (2 n = 2 x = 34), which was the same as that of the controls; thus, the univalent was observed during PMC meiosis was not caused by an unbalanced chromosome number. Then, we analyzed the chromosome karyotype. The relative length (%) (long arm + short arm = full length), arm ratio (long/short) and chromosome type were assessed (Supplementary Table 4). The karyotype formulas were as follows: 2n = 2x = 26 m + 8 sm (H30-6), 2n = 2x = 18 m + 16 sm (H411), and 2n = 2x = 30 m + 4 sm (B336). The chromosome lengths varied from 1.54 ± 0.08-2.63 ± 0.09 μm (H30-6), 2.02 ± 0.16–3.59 ± 0.16 μm (H411), and 1.57 ± 0.05–2.5 ± 0.1 μm (B336). According to the chromosome field theory, the chromosomes belonged to the small chromosome. The ratios of the longest chromosome to the shortest were 1.71 (H30-6), 1.78 (H411), and 1.59 (B336), and chromosome arm ratios above 2 accounted for 0.03 (H30-6), 0.13 (H411), and 0.06 (B336) of the total ratios. Therefore, according to Stebbins's (1971) classification, the karyotypes were all 2A types. The asymmetrical karyotype coefficients were 59.03% (H30-6), 61.95% (H411), and 59.23% (B336). Figure 8 shows the chromosome images, karyograms, and idiograms of H30-6, H411, and B336, respectively. The chromosome observation results revealed no obvious chromosomal numerical or structural abnormalities.

## Discussion

### Low Female Gametophyte Fertility Is Mainly Responsible for the Low Seed Formation of H30-6

Few-seed usually results from low gametophyte fertility (Xiao et al., 2007; Rédei, 2008; Qiu et al., 2012; Li et al., 2016; Royo et al., 2016), self-incompatibility (Zhang et al., 2012; Gambetta et al., 2013; Garmendia et al., 2019) and embryo abortion (Pearson, 1932; Tang, 2001). There was no self-incompatibility found for H30-6, which can be demonstrated proofed by the result of artificial selfing and *in situ* pollen germination. As a result, low fertility should be responsible for the few-seed nature of H30-6. There were no abnormalities found in flower morphology, but only limited pollen existed on the dehiscent anther indicating a low male gamete fertility character. Additionally, this was verified according to the vertical sections of mature anther and pollen morphology observation. Further, the pollen viability test showed that only 4.04% of pollen could germinate *in vitro*. So H30-6 was a male sterile material depending on Deng (2009a) standard. However, the pollination treatment results showed that there was no significant difference in the seed number (1.59-2) per fruit when the pollen of H30-6, H411, and B336 was used or open-pollinated. Meanwhile, the former embryo sac observation results showed that there were only 2.25 normal ovules in each ovary of H30-6. It was reasonable to assume that abnormal embryo sacs should be responsible for low seed formation.

### May Abnormal Meiosis Lead to Low Female Gametophyte Fertility of H30-6?

The formation of both male and female gametophytes is a precisely coordinated process, including the formation of gametophyte mother cells, meiosis, and gametophyte development. Therefore, there are three likely mechanisms leading to low gametophyte fertility: pre meiosis abnormality, such as a lack of sporogenous cells (Schiefthaler et al., 1999; Yang et al., 1999) and defects in meiotic entry (Hong et al., 2012), meiosis abnormalities, such as arrests at one meiosis period (Siddiqi et al., 2000) and aberrant chromosome distribution (Hoyt and Geiser, 1996; d'Erfurth et al., 2008; De Storme and Geelen, 2011), and postmeiotic abnormality, especially reproductive companion cell abnormalities, which will cause gametophytes to be sterile (Chaubal et al., 2000; Guo et al., 2001; Suzuki et al., 2001; Smith et al., 2002; Kawanabe et al., 2006; Li et al., 2006; Zhang et al., 2006). In this study, we found no premeiotic or post-meiotic abnormalities but a multitude of meiosis abnormal phenomena of PMCs. Therefore, low male fertility is caused by abnormal meiosis. Although we did not observe the meiosis of megasporocytes, the differentiation shutoff or degradation of female gametes showed that most of the functional macrospores were disabled, which we speculated could be due to that there were meiotic abnormalities in megasporocytes.

### Possible Mechanism of H30-6 Abnormal Meiosis

Chromosome number and structural changes can lead to abnormal meiosis (Grandont et al., 2014; Rocha et al., 2019). Low fertility is often found in polyploids, which have been used for loquat seedless and few-seed breeding (Maria et al., 2013). H30-6 was identified as being a diploid line. That was to say, the abnormal meiosis of H30-6 was not caused by polyploidization. A recent report showed that a 2.09 Mb chromosome fragment translocation causes abnormalities during meiosis and leads to pollen semisterility and fewer seeds in watermelon (Tian et al., 2021). Additionally, the fragment translocation results in one quadrivalent ring of four chromosomes at prometaphase I during meiosis. During the meiosis of H30-6, the earliest abnormalities were found during diakinesis, and a high frequency of univalent chromosomes (6.87) was the main abnormality. The most likely reason was that homolog pairing failure caused abnormal meiosis.

Homolog pairing is subdivided into three levels: preselection of homologous chromosomes, homologous connections by the synaptonemal complex (the process also known as synapsis), and the precise matching of DNA molecules in the process of genetic exchange and conversion (the process also known as gene recombination) (Moens, 1969; Loidl, 1990). Abnormalities occurring in all three of these levels may result in the univalent characteristics found in diakinesis.

When or how homologous chromosomes select each other is still unknown (Bozza and Pawlowski, 2008); However, chromosomal movements that are performed prior to and as a precondition to any homologous interaction have been widely supported (Loidl, 1990; Dawe et al., 1994; Bass, 1997), especially when considering telomere mediated movement (Lange, 1998; Nimmo et al., 1998; Trelles-Sticken and Scherthan, 2000; Sepsi and Schwarzacher, 2020). Varas et al. (2015) reported that two Sad1/UNC-84 (SUN)-domain proteins (AtSUN1 and AtSUN2) play an important role in telomeres movement during meiosis, and the double mutant exhibited severe meiotic defects, including a delay in the progression of meiosis, an absence of full synapsis, and a reduction in the mean cell chiasma frequency.

The synaptonemal complex stabilizes the presynaptic alignment of the axes of the homologous chromosomes and promotes the maturation of crossover recombination events, which is widely conserved in sexually reproducing eukaryotes with the lateral elements (LEs), central elements (CEs), and transverse element (transverse filaments, TFs) (Gillies and Moens, 1984; Christa, 1996; Dawe, 1998). Thus far, several proteins involved in LE, such as budding yeast Hop1 and Red1; mice SYCP2 and SYCP3; nematode HIM-3, HTP-1, HTP-2, and HTP-3; and plants proteins ASY1/PAIR2 and ASY3/PAIR3 have been reported (Hollingsworth et al., 1990; Smith and Roeder, 1997; Yang et al., 2006; Sanchez-Moran et al., 2008; Ferdous et al., 2012; Lui and Colaiacovo, 2013). Furthermore, yeast Zip1, mice SYCP1, Drosophila C (3) G, nematode SYP-1/2/3/4, Arabidopsis thaliana ZYP1, and rice ZEP1 have been reported to be components of TFs (Sym et al., 1993; Page and Hawley, 2001; De Vries et al., 2005; Higgins et al., 2005; Wang et al., 2010; Lui and Colaiacovo, 2013).

The gene recombination structure, which is also known as the visualized crossover (CO), links the homologs after diplotene in most organisms. Typically, every chromosome undergoes at least one obligate CO event to ensure proper segregation at metaphase I (Jones, 1984). The double-strand break repair model (DSBR) explained the gene recombination (Szostak et al., 1983; Mercier et al., 2015). Meiotic DNA DSBs are formed by the highly conserved SPO11 protein (Edlinger and Schlogelhofer, 2011; Bernard, 2013) and PRD1, PRD2, AtPRD3/OsPAIR1, DFO, and CRC1 proteins in plants (De Muyt et al., 2009; Miao et al., 2013). In most species, homologous chromosomes do not pair properly in mutants that do not normally form meiotic DNA double-strand-breaks (DSBs) (Peoples-Holst and Burgess, 2005; Yu et al., 2010; Ji et al., 2016; Robert et al., 2016). The plant MRE11, RAD50, and COM1 genes are functionally conserved in the early steps of DSB processing, and their respective mutants all display strong chromosome fragmentation at anaphase I (Mercier et al., 2015). In Arabidopsis, DMC1-mediated inter-homologous (IH) DNA repair is the predominant pathway of DSB repair, and when DMC1 is missing, RAD51 works as a backup pathway leading to a complete absence of synapsis and bivalent formation (Couteau et al., 1999; Deng and Wang, 2007). Finally, the CO formation relies on two pathways: the ZMM pathway, which relies on a group of proteins called ZMMs, as well as proteins MLH1 and MLH3, and the non-ZMM CO pathway, in which protein MUS81 has been characterized in the non-ZMM CO pathway in plants (Mercier et al., 2015). The mutants of the ZMM pathway and MUS81 were reported to exhibit various degrees of decrease in COs (Mercier et al., 2015).

Therefore, to clarify the molecular mechanism of H30-6 abnormal meiosis, the above proteins involved in the homolog pairing constitute the main points of this study.

## Conclusions

The diploid loquat line H30-6 is a potentially commercial few-seed (1.54 per fruit) loquat, with high fruit edible rate (70.77%). The diploid loquat line H30-6 is a male sterile material, and this sterility is caused by the abnormal meiotic synapses. The low fertility of H30-6 pollen was a limiting factor for the fruit setting. The main reason for the few seeds character of H30-6 was the female gamete abnormality.

## Data Availability Statement

The original contributions presented in the study are included in the article/Supplementary Material, further inquiries can be directed to the corresponding authors.

## Author Contributions

GL, QG, and JD conceived and designed the research and revised the manuscript. QX performed the experiments and wrote and revised the manuscript. XW and SX helped with the data analysis and revised the manuscript. PW and SL managed the material collection. XL and QH helped with the material filed management. HS, DW, YX, SW, and DJ helped with the fund management, drug purchase, and instrument management. All the authors read and approved the final manuscript.

## Funding

This research was funded by the National Key R&D Program of China (No. 2019YFD1000900), the National Nature Science Foundation of China (32171820), the Chongqing Science and Technology Commission (cstc2021jscx-gksbX0010 and cstc2021jcyj-sxmX1156), the Innovation Research Group Funds for Chongqing Universities (CXQT19005), and the Chongqing Forestry Administration (YuLinKeYan2022-14).

## Conflict of Interest

The authors declare that the research was conducted in the absence of any commercial or financial relationships that could be construed as a potential conflict of interest.

## Publisher's Note

All claims expressed in this article are solely those of the authors and do not necessarily represent those of their affiliated organizations, or those of the publisher, the editors and the reviewers. Any product that may be evaluated in this article, or claim that may be made by its manufacturer, is not guaranteed or endorsed by the publisher.

## References

[B1] Arano H.. (1963). Cytological studies in subfamily Carduoideae (compositae) of Japan IX. Bot. Mag. Tokyo. 76, 32–39. 10.15281/jplantres1887.76.32

[B2] Bass H. W.. (1997). Telomeres cluster *de novo* before the initiation of synapsis: a three-dimensional spatial analysis of telomere positions before and during meiotic prophase. J. Cell Biol. 137, 5–18. 10.1083/jcb.137.1.5 9105032 PMC2139864

[B3] Bernard D. M.. (2013). Initiation of meiotic recombination: how and where? conservation and specificities among eukaryotes. Annu. Rev. Genet. 47, 563–599. 10.1146/annurev-genet-110711-155423 24050176

[B4] Bozza C. G. Pawlowski W. P. (2008). The cytogenetics of homologous chromosome pairing in meiosis in plants. Cytogenet. Genome Res. 120, 313–319. 10.1159/000121080 18504360

[B5] Chaubal R. Zanella C. Trimnell M. R. Fox T. W. Bedinger A. P. (2000). Two male-sterile mutants of Zea Mays (Poaceae) with an extra cell division in the anther wall. Am. J. Bot. 87, 1193–1201. 10.2307/2656657 10948005

[B6] Chen W. Feng J. Qin Q. Liu X. Wu J. Xie M. (2006). Characteristics of sugar metabolism and accumulation in GA_3 induced parthenocarpic white flesh Loquat ‘Ninghai Bai' Fruit. Acta Hortic. Sin. 33, 471–476. 10.16420/j.issn.0513-353x.2006.03.004

[B7] Christa H.. (1996). Synaptonemal complexes: structure and function. Curr. Opin. Cell Biol. 8, 389–396. 10.1016/S0955-0674(96)80015-9 8743892

[B8] Couteau F. Belzile F. Horlow C. Grandjean O. Vezon D. Doutriaux M. P.. (1999). Random chromosome segregation without meiotic arrest in both male and female meiocytes of a *dmc1* mutant of *Arabidopsis*. Plant Cell. 11, 1623–1634. 10.1105/tpc.11.9.1623 10488231 PMC144309

[B9] Dafni A. Kevan P. G. Husband B. C. (2005). Practical Pollination Biology. Cambridge: Enviroquest Ltd. p, 120–121.

[B10] Dang J. Guo Q. Xiang S. He Q. Sun H. Wu D.. (2019a). A New Red-fleshed and Seedless Loquat Cultivar with Large Fruit ‘Huajin Wuhe No. 1'. Acta Hortic. Sin. 46, 2763–2764. 10.16420/j.issn.0513-353x.2019-0241

[B11] Dang J. Guo Q. Xiang S. He Q. Sun H. Wu D.. (2019b). A New Seedless Loquat Cultivar ‘Huayu Wuhe No. 1' with Large Fruit. Acta Hortic. Sin. 2776–2767. 10.16420/j.issn.0513-353x.2019-0242

[B12] Dawe R. K.. (1998). Meiotic chromosome organization and segregation in plants. Annu. Rev. Plant Biol. 49, 371–395. 10.1146/annurev.arplant.49.1.371 15012239

[B13] Dawe R. K. Sedat J. W. Agard D. A. Cande W. Z. (1994). Meiotic chromosome pairing in maize is associated with a novel chromatin organization. Cell. 76, 901–912. 10.1016/0092-8674(94)90364-6 8124724

[B14] De Muyt A. Pereira L. Vezon D. Chelysheva L. Gendrot G. Chambon A.. (2009). A high throughput genetic screen identifies new early meiotic recombination functions in *Arabidopsis thaliana*. PLOS Genetics. 5, e1000654. 10.1371/journal.pgen.1000654 19763177 PMC2735182

[B15] De Storme N. Geelen D. (2011). The arabidopsis mutant jason produces unreduced first division restitution male gametes through a parallel/fused spindle mechanism in meiosis II. Plant Physiol. 155, 1403–1415. 10.1104/pp.110.170415 21257792 PMC3046594

[B16] De Vries F. A. T. de Boer E. van den Bosch M. Baarends W. M. Ooms M. Yuan L.. (2005). Mouse Sycp1 functions in synaptonemal complex assembly, meiotic recombination, and XY body formation. Genes Dev. 19, 1376–1389. 10.1101/gad.329705 15937223 PMC1142560

[B17] Deng Q.. (2009a). Studies on Embryological Mechanism of Seed Degeneration and Genetic Diversity of Seedlings from Middle-Degenerated Seeds in Loquat (*Eriobotrya japonica* Lindl.). [dissertation/Doctoral thesis]. Sichuan: Sichuan Agricultural University.

[B18] Deng X. Guo W. Sun X. (1996). Research progress on the type selection of seedless citrus in China literature review. Acta Hortic. Sin. 23, 235–240.

[B19] Deng Y.. (2009b). Research on the Nucle-free induction mechanism of GA3 in Eriobotrya japonica. [Dissertation/doctoral thesis]. Chongqing: Southwest University.

[B20] Deng Z. Wang T. (2007). OsDMC1 is required for homologous pairing in *Oryza sativa*. Plant Mol. Biol. 65, 31–42. 10.1007/s11103-007-9195-2 17562186

[B21] d'Erfurth I. Jolivet S. Froger N. Catrice O. Novatchkova M. Simon M.. (2008). Mutations in AtPS1 (*Arabidopsis thaliana* Parallel Spindle I) Lead to the Production of Diploid Pollen Grains. PLOS Genetics. 4:e1000274. 10.1371/journal.pgen.1000274 19043546 PMC2581889

[B22] Dong M. Li J. Zhou D. Yue J. Gao J. (2013). Advances in citrus cultivars breeding. Chinese Fruit Trees. p, 73–78.

[B23] Edlinger B. Schlogelhofer P. (2011). Have a break: determinants of meiotic DNA double strand break (DSB) formation and processing in plants. J. Exp. Bot. 62, 1545–1563. 10.1093/jxb/erq421 21220780

[B24] Fan P. Yang M. Zhang Y. Li S. (2004). Early-ripening Seedless Grape ‘Jingzaojing'. Acta Hortic. Sin. 31, 415. 10.16420/j.issn.0513-353x.2004.03.036

[B25] Ferdous M. Higgins J. D. Osman K. Lambing C. Roitinger E. Mechtler K.. (2012). Inter-homolog crossing-over and synapsis in *Arabidopsis* meiosis are dependent on the chromosome axis protein AtASY3. PLOS Genet. 8, e1002507. 10.1371/journal.pgen.1002507 PMC3271061 22319460

[B26] Gambetta G. Gravina A. Fasiolo C. Fornero C. Galiger S. Inzaurralde C.. (2013). Self-incompatibility, parthenocarpy and reduction of seed presence in ‘afourer' mandarin. Sci. Hortic. 164, 183–188. 10.1016/j.scienta.2013.09.002

[B27] Garmendia A. Beltrán R. Zornoza C. Breijo F. Reig J. Bayona I.. (2019). Insect repellent and chemical agronomic treatments to reduce seed number in 'afourer' mandarin. effect on yield and fruit diameter. Sci. Hortic. 246, 437–447. 10.1016/j.scienta.2018.11.025

[B28] Gillies C. B. Moens P. B. (1984). The synaptonemal complex in higher plants. Crit. Rev. Plant Sci. 2, 81–116. 10.1080/07352688409382191

[B29] Grandont L. Cunado N. Coriton O. Huteau V. Eber F. Chevre A. M.. (2014). Homoeologous chromosome sorting and progression of meiotic recombination in brassica napus: ploidy does matter! *Plant Cell*. 26, 1448–1463. 10.1105/tpc.114.122788 24737673 PMC4036564

[B30] Gu J. Zhang S. (1990). Production of seedless loquat. Foreign agriculture. 3, 31–33.

[B31] Guo J. Sun R. Song J. Zhang S. (2001). Microsporogenesis of Several-Male 2 Sterile Lines in Brassica rapa L. ssp *pekinensis*. Acta Hortic. Sin. 28, 409–414. 10.3321/j.issn:0513-353X.2001.05.005

[B32] Guo Q. Li X. Xiang S. He Q. Sun H. Wu D.. (2016). A new white pulp seedless loquat cultivar ‘Wuhe Guoyu'. Acta Hortic. Sin. 43, 2717–2718. 10.16420/j.issn.0513-353x.2016-0445

[B33] Higgins J. D. Sanchez-Moran E. Armstrong S. J. Jones G. H. Franklin F. C. H. (2005). The *Arabidopsis* synaptonemal complex protein ZYP1 is required for chromosome synapsis and normal fidelity of crossing over. Genes Dev. 19, 2488–2500. 10.1101/gad.354705 16230536 PMC1257403

[B34] Hollingsworth N. M. Goetsch L. Byers B. (1990). The hop1 gene encodes a meiosis-specific component of yeast chromosomes. Cell. 61, 73–84. 10.1016/0092-8674(90)90216-2 2107981

[B35] Hong L. Tang D. Zhu K. Wang K. Li M. Cheng Z. (2012). Somatic and reproductive cell development in rice anther is regulated by a putative glutaredoxin. Plant Cell. 24, 577–588. 10.1105/tpc.111.093740 22319054 PMC3315234

[B36] Hoyt M. A. Geiser J. R. (1996). Genetic analysis of the mitotic spindle. Annu. Rev. Genet. 30:7–33. 10.1146/annurev.genet.30.1.7 8982447

[B37] Hu S.. (1982). Angiosperms Embryology. Beijing: Higher Education Press. p, 54.

[B38] Hu S.. (1993). Experimental methods in plant embryology (I) determination of pollen viability. Chin. J. Bot. 10, 60–62.

[B39] Huang J. Xu X. (1980). Loquat new varieties ‘TaiCheng No.4'. China Fruits. 1, 35–37.

[B40] Huang J. Xu X. Chen X. (1984). Cultivation of tetraploid loquat ‘Min NO.3'. Chinese Fruit Trees. p, 27–30.

[B41] Ji J. Tang D. Shen Y. Xue Z. Wang H. Shi W. Q.. (2016). P31comet, a member of the synaptonemal complex, participates in meiotic DSB formation in rice. Proc. Natl. Acad. Sci. U.S.A. 113, 10577–10582. 10.1073/pnas.1607334113 27601671 PMC5035842

[B42] Jiang F. Huang A. Chen Z. Deng C. Chen X. Chen X.. (2009). Study on seed traits in Loquat (Eriobotrya Japonica Lindl.) germplasm resources. *Fujian*. Hortic. 4, 19–24. 10.3969/j.issn.1004-6089.2009.04.005

[B43] Jones G. H.. (1984). The control of chiasma distribution. Symp. Soc. Exp. Biol. 38, 293–320. 6545727

[B44] Kawanabe T. Ariizumi T. Kawai-Yamada M. Uchimiya H. Toriyama K. (2006). Abolition of the tapetum suicide program ruins microsporogenesis. Plant Cell Physiol. 47, 784–787. 10.1093/pcp/pcj039 16565524

[B45] Kikuchi S. Iwasuna M. Kobori A. Tsutaki Y. Yoshida A. Murota Y.. (2014). Seed formation in triploid loquat (Eriobotrya japonica) through cross-hybridization with pollen of diploid cultivars. Breed. 64,176–182. 10.1270/jsbbs.64.176 PMC4065325 24987304

[B46] Lange T. D.. (1998). Telomeres and senescence: ending the debate. Science. 279, 334–335. 10.1126/science.279.5349.334 9454329

[B47] Ledbetter C. A. Ramming D. W. (1989). “Seedlessness in Grapes”, in *Horticultural Reviews II*, ed. J. Janick (Portland: Timber Press). p, 159–184. 10.1002/9781118060841.ch5

[B48] Levan A. Fredga K. Sandberg A. A. (1964). Nomenclature for centromeric position of chromosomes. Hereditas. 52, 201–220. 10.1111/j.1601-5223.1964.tb01953.x

[B49] Li M. Chen R. (1985). A suggestion on the standardization of karyotype analysis in plants. *J*. Wuhan Bot. Res. 3, 297–302.

[B50] Li M. Lu C. Liu X. Wang X. Zheng X. (2016). Research progress of seedless and stenospermocarpic mechanism in litchi. J. Trop. Crop Sci. 37, 7. 10.3969/j.issn.1000-2561.2016.05.030

[B51] Li N. Zhang D. Liu H. Yin C. Zhang D. (2006). The rice tapetum degeneration retardation gene is required for tapetum degradation and anther development. Plant Cell. 18, 2999–3014. 10.1105/tpc.106.044107 17138695 PMC1693939

[B52] Li S. Wang Y. (2019). Advances in seedless gene researches and seedless breeding in grapevine. Acta Hortic. Sin. 9, 16. 10.16420/j.issn.0513-353x.2019-0035

[B53] Li Z.. (1987). Plant Section Technology. Beijing: Science Press.

[B54] Li Z. Luo Q. Wang Y. (2019). Breeding seedless grapevine *via* embryo rescue and marker-assisted selection in hybrid progenies. J. Fruit Sci. 36, 12. 10.13925/j.cnki.gsxb.20180098

[B55] Liang G. Li X. (1991). Improved smear method of plant meiosis on citrus. South China Fruits. 2, 40–41.

[B56] Liang S. Dang J. Liang G. Guo Q. (2018). Meiosis Observation and Fertility Analysis in Natural Tetraploid Loquat of ‘B431'. Acta Hortic. Sin. 45, 1895–1904. 10.16420/j.issn.0513-353x.2018-0222

[B57] Lin S.. (2008). “Loquat,” in: *Encyclopedia of Fruits and Nuts* (Wallingford: CABI). p, 643–651.

[B58] Lin S. Sharpe R. H. Janick J. (1999). Loquat: Botany and horticulture. Hortic. Rev. 23, 233–276. 10.1002/9780470650752.ch534910340

[B59] Loidl J.. (1990). The initiation of meiotic chromosome pairing: the cytological view. Genome. 33, 759. 10.1139/g90-115 2086352

[B60] Lui D. Y. Colaiacovo M. P. (2013). Meiotic development in Caenorhabditis elegans. Adv. Exp. Med. Biol. 757, 133–170. 10.1007/978-1-4614-4015-4_6 22872477 PMC3764601

[B61] Maria L. Janick J. Lin S. Wang W. Liang G. Wang W. (2013). “Breeding Loquat”, in Plant Breeding Reviews, ed. by J. Janick, (New Jersey, NJ: John Wiley and Sons, Inc.). p, 259–296. 10.1002/9781118497869.ch5

[B62] Mercier R. Mézard C. Jenczewski E. Macaisne N. Grelon M. (2015). The molecular biology of meiosis in plants. Annu. Rev. Plant Biol. 66, 297–327. 10.1146/annurev-arplant-050213-035923 25494464

[B63] Miao C. Tang D. Zhang H. Wang M. Li Y. Tang S.. (2013). Central region component1, a novel synaptonemal complex component, is essential for meiotic recombination initiation in rice. Plant Cell. 25, 2998–3009. 10.1105/tpc.113.113175 23943860 PMC3784594

[B64] Moens P. B.. (1969). The fine structure of meiotic chromosome polarization and pairing in locusta migratoria spermatocytes. Chromosoma. 28, 1–25. 10.1007/BF00325986 5365542

[B65] Nimmo E. R. Pidoux A. L. Perry P. E. Allshire R. C. (1998). Defective meiosis in telomere-silencing mutants of schizosaccharomyces pombe. Nature. 392, 825–828. 10.1038/33941 9572142

[B66] Page S. L. Hawley R. S. (2001). c (3)G encodes a *Drosophila* synaptonemal complex protein. Gene Dev. 15, 3130–3143. 10.1101/gad.935001 11731477 PMC312841

[B67] Pearson H. M.. (1932). Parthenocarpy and seedlessness in *Vitis vinifera*. Science. 76, 594–594. 10.1126/science.76.1982.594.a 17842446

[B68] Peoples-Holst T. L. Burgess S. M. (2005). Multiple branches of the meiotic recombination pathway contribute independently to homolog pairing and stable juxtaposition during meiosis in budding yeast. Gene Dev. 19, 863–874. 10.1101/gad.1293605 15805472 PMC1074323

[B69] Qiu W. Zhu A. Wang Y. Chai L. Ge X. Deng X.. (2012). Comparative transcript profiling of gene expression between seedless ponkan mandarin and its seedy wild type during floral organ development by suppression subtractive hybridization and cdna microarray. BMC Genomics. 13, 397. 10.1186/1471-2164-13-397 PMC3495689 22897898

[B70] Rédei G. P.. (2008). Encyclopedia of genetics, genomics, proteomics, and informatics. Springer Science and Business Media. Volume 1 A-L, p. 1773. 10.1007/978-1-4020-6754-9

[B71] Robert T. Nore A. Brun C. Maffre C. Crimi B. Bourbon H. M.. (2016). The TopoVIB-Like protein family is required for meiotic DNA double-strand break formation. Science. 351, 943–949. 10.1126/science.aad5309 26917764

[B72] Rocha M. Chiavegatto R. B. Damasceno A. G. Rocha L. C. Techio V. H. (2019). Comparative meiosis and cytogenomic analysis in euploid and aneuploid hybrids of *Urochloa* P. beauv. Chromosome Res. 27, 333–344. 10.1007/s10577-019-09616-y 31485871

[B73] Royo C. Carbonell-Bejerano P. Torres-Pérez R. Nebish A. Martínez Ó. Rey M.. (2016). Developmental, transcriptome, and genetic alterations associated with parthenocarpy in the grapevine seedless somatic variant Corinto bianco. J. Exp. Bot. 67:259–273. 10.1093/jxb/erv452 26454283

[B74] Sanchez-Moran E. Osman K. Higgins J. D. Pradillo M. Cuñado N. Jones G. H.. (2008). ASY1 coordinates early events in the plant meiotic recombination pathway. Cytogenet. Genome Res. 120, 302–312. 10.1159/000121079 18504359

[B75] Schiefthaler U. Balasubramanian S. Sieber P. Chevalier D. Wisman E. Schneitz K. (1999). Molecular analysis of nozzle, a gene involved in pattern formation and early sporogenesis during sex organ development in Arabidopsis thaliana. in: *Proceedings of the National Academy of Sciences of the United States of America* (Washington, DC). 96, 11664–11664. 10.1073/pnas.96.20.11664 10500234 PMC18091

[B76] Sepsi A. Schwarzacher T. (2020). Chromosome-nuclear envelope tethering-a process that orchestrates homologue pairing during plant meiosis? J. Cell Sci. 133:jcs243667. 10.1242/jcs.243667 32788229 PMC7438012

[B77] Siddiqi I. Ganesh G. Grossniklaus U. Subbiah V. (2000). The dyad gene is required for progression through female meiosis in arabidopsis. Development. 127, 197–207. 10.1242/dev.127.1.197 10654613

[B78] Smith A. V. Roeder G. S. (1997). The Yeast Red1 Protein Localizes to the Cores of Meiotic Chromosomes. J. Cell Biol. 136, 957–967. 10.1083/jcb.136.5.957 9060462 PMC2132480

[B79] Smith M. B. Palmer R. G. Horner H. T. (2002). Microscopy of a cytoplasmic male-sterile soybean from an interspecific cross between Glycine max and *G*. soja (Leguminosae). *Am. J. Bot*. 89, 417–417. 10.3732/ajb.89.3.417 21665637

[B80] Stebbins G. L.. (1971). Chromosomal evolution in higher plants. London: Arnold. p, 216.

[B81] Suzuki K. Takeda H. Tsukaguchi T. Egawa Y. (2001). Ultrastructural study on degeneration of tapetum in anther of snap bean (*Phaseolus vulgaris L*.) under heat stress. Sex. Plant Reprod. 13, 293–299. 10.1007/s004970100071

[B82] Sym M. Engebrecht J. Roeder G. S. (1993). Zip1 is a synaptonemal complex protein required for meiotic chromosome synapsis. Cell. 72, 365–378. 10.1016/0092-8674(93)90114-6 7916652

[B83] Szostak J. W. Orr-Weaver T. L. Rothstein R. J. Stahl F. W. (1983). The double-strand-break repair model for recombination. Cell. 33, 25–35. 10.1016/0092-8674(83)90331-8 6380756

[B84] Tang X.. (2001). Study on seedless or less seedless mechanism and seedless type cultivation of persimmon. [dissertation/doctoral thesis]. Wuhan: Huazhong Agricultural University.

[B85] Tian S. Ge J. Ai G. Jiang J. Liu Q. Chen X.. (2021). A 2.09 Mb fragment translocation on chromosome 6 causes abnormalities during meiosis and leads to less seed watermelon. Hortic. Res. 8:256. 10.1038/s41438-021-00687-9 34848689 PMC8633341

[B86] Trelles-Sticken E. Scherthan D. H. (2000). Meiotic telomere protein ndj1p is required for meiosis-specific telomere distribution, bouquet formation and efficient homologue pairing. J. Cell Biol. 151, 95–106. 10.1083/jcb.151.1.95 11018056 PMC2189801

[B87] Varas J. Graumann K. Osman K. Pradillo M. Evans D. E. Santos J. L.. (2015). Absence of sun1 and sun2 proteins in *Arabidopsis thaliana* leads to a delay in meiotic progression and defects in synapsis and recombination. Plant J. 81, 329–346. 10.1111/tpj.12730 25412930

[B88] Wang H. Dang J. Wu D. Xie Z. Yan S. Luo J.. (2021). Genotyping of polyploid plants using quantitative PCR: application in the breeding of white-fleshed triploid loquats (Eriobotrya japonica). Plant Methods. 17, 93. 10.1186/s13007-021-00792-9 PMC8418031 34479588

[B89] Wang M. Wang K. Tang D. Wei C. Li M. Shen Y.. (2010). The central element protein ZEP1 of the synaptonemal complex regulates the number of crossovers during meiosis in rice. Plant Cell. 22, 417–430. 10.1105/tpc.109.070789 20154151 PMC2845403

[B90] Xiao J. Tan J. Liu H. Chen L. Ye W. Cheng W. (2007). Studies on the seedless mechanism of ‘Lipeng No.2' ponkan (Citrus reticulata). J. Fruit Sci. 4, 421–426. 10.13925/j.cnki.gsxb.2007.04.003

[B91] Xu F. Zhang,. X Shi,. C Wang,. X Xi,. X Jiang,. S. (2016). A study on the influence factors of single-seeded formation in loquat. J. Fruit Sci. 33, 676–685. 10.13925/j.cnki.gsxb.20160099

[B92] Xu X. Si J. Xie J. Lan Y. Zeng Y. Jiang W.. (2006). Ougan seedless, a new mandarin cultivar. J. Fruit Trees. 5, 781–782. 10.13925/j.cnki.gsxb.2006.05.031

[B93] Yang F. Fuente R. Leu N. A. Baumann C. McLaughlin K. J. Wang P. (2006). Mouse SYCP2 is required for synaptonemal complex assembly and chromosomal synapsis during male meiosis. J. Cell Biology. 173, 497–507. 10.1083/jcb.200603063 16717126 PMC2063860

[B94] Yang W. Ye D. Xu J. Sundaresan V. (1999). The SPOROCYTELESS gene of Arabidopsis is required for initiation of sporogenesis and encodes a novel nuclear protein. Genes Dev. 13, 2108–2117. 10.1101/gad.13.16.2108 10465788 PMC316961

[B95] Yang Y.. (2021). Analysis of female fertility of triploid loquat (Eriobotrya japonica) Q24 and preliminary study on genome characteristics. [dissertation/master's thesis]. Chongqing: Southwest University.

[B96] Ye Z. Zeng T. Xu J. Luo Z. Hu G. Zhang Z.. (2006). Breeding of seedless tangerine (shiyutangerine). J. Fruit Sci. 23,149–150+F0002. 10.1016/j.compscitech.2006.07.013

[B97] Yu H. Wang M. Tang D. Wang K. Chen F. Gong Z.. (2010). OsSPO11-1 is essential for both homologous chromosome pairing and crossover formation in rice. Chromosoma. 119, 625–636. 10.1007/s00412-010-0284-7 20625906

[B98] Zhang G. Kang L. Gao Z. Zhu S. Gao K. (1999). Effects of Ga and CPPU on quality of loquat seedless fruit. J. Fruit Sci. 16, 55–59.

[B99] Zhang S. Huang G. Ding F. He X. Pan J. (2012). Mechanism of seedlessness in a new lemon cultivar ‘Xiangshui' [Citrus limon (L.) Burm. F.]. Sex. Plant Reprod. 25:337–345. 10.1007/s00497-012-0201-8 23114638

[B100] Zhang W. Sun Y. Timofejeva L. Chen C. Ma H. (2006). Regulation of Arabidopsis tapetum development and function by Dysfunctional Tapetum1 (DYT1) encoding a putative bHLH transcription factor. Development. 133, 3085–3095. 10.1242/dev.02463 16831835

[B101] Zhang X. Liao M. He J. Liu C. Ma Q. Yang D.. (2015). Study on Stigma Receptivity and Pollen Tube growth of 'Chuanzao loquat' in the First Florescence. Acta Bot. Sin. 1349–1355. 10.7606/j.issn.1000-4025.2015.07.1349

[B102] Zhang X. Liao M. Wang Y. Li X. Tao L. (2014). Research on the Difference in the Pollen Tube Growth of a Low Seediness Line of Loquat. Acta Bot. Sin. 34, 26–31. 10.7606/j.issn.1000-4025.2014.01.0026

[B103] Zilli A. L. Brugnoli E. A. Marcón F. Billa M. B. Rios E. F. Martínez E. J.. (2015). Heterosis and expressivity of apospory in tetraploid bahiagrass hybrids. Crop Sci. 55, 1189–1201. 10.2135/cropsci2014.10.0685

