# Supplementary material for: Low Female Gametophyte Fertility Contributes to the Low Seed Formation of the Diploid Loquat [Eriobotrya Japonica (Thunb.) Lindl.] Line H30-6
Source: Front Plant Sci. 2022 May 23;13:882965. doi: 10.3389/fpls.2022.882965 (PMC9168767; doi:10.3389/fpls.2022.882965)
Supplement: Supplementary file 1 [file Data_Sheet_1.PDF]

Supplementary Material

Supplementary Figures

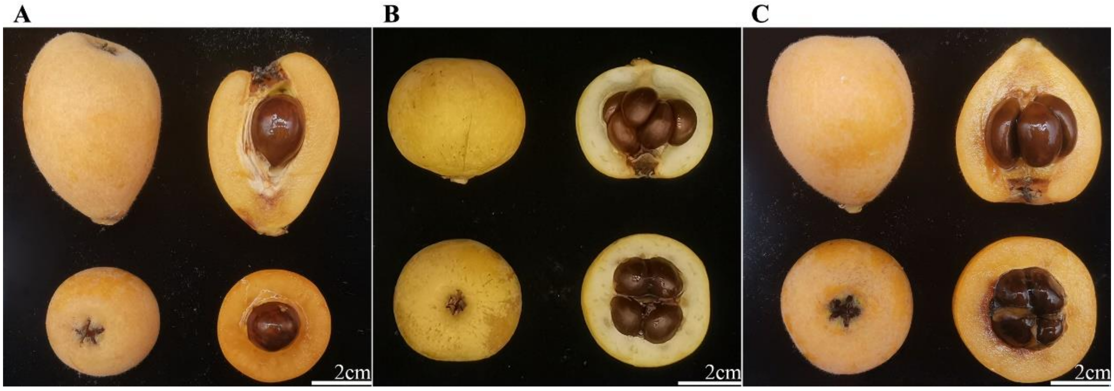

Supplementary Figure 1. Images of fruit and fruit transverse and longitudinal sections: (A) H30-6, (B) H411, (C) B336.

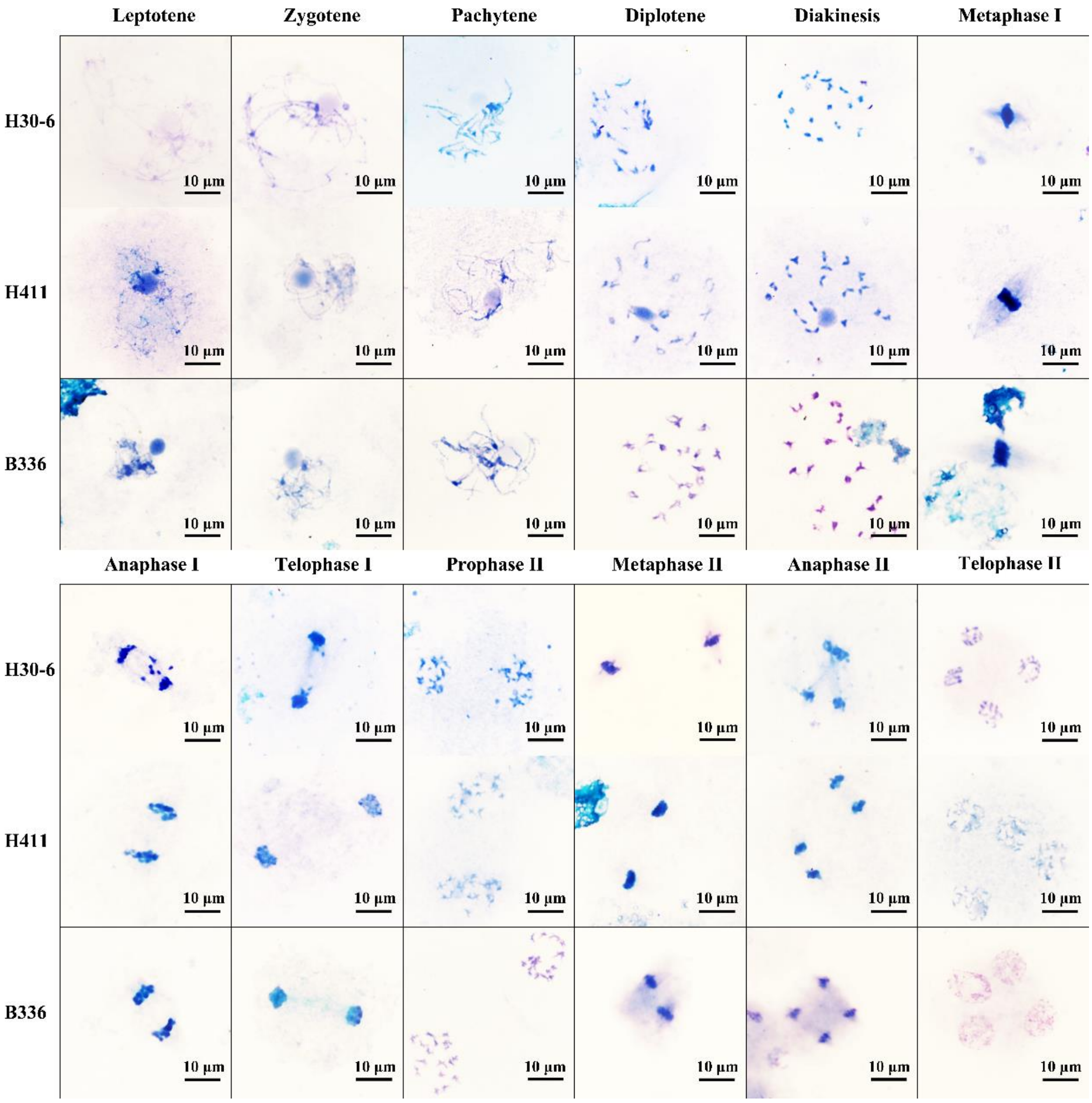

Supplementary Figure 2. MMCs meiosis process of H30-6, H411 and B336 respectively.

Supplementary Tables

Supplementary Table 1. Chromosome configuration at diakinesis

MMC NO.	H30-6					MMC NO.	H411				MMC NO.	B336	
	I	II	III	IV	V		I	II	III	IV		I	II
1	32	1	0	0	0	1	2	16	0	0	1	2	16
2	7	9	3	0	0	2	0	17	0	0	2	0	17
3	8	7	0	3	0	3	0	17	0	0	3	0	17
4	11	7	3	0	0	4	0	17	0	0	4	0	17
5	13	7	1	1	0	5	0	17	0	0	5	0	17
6	34	0	0	0	0	6	0	17	0	0	6	0	17
7	34	0	0	0	0	7	0	17	0	0	7	0	17
8	5	10	3	0	0	8	2	16	0	0	8	0	17
9	22	6	0	0	0	9	2	16	0	0	9	0	17
10	0	17	0	0	0	10	2	16	0	0	10	0	17
11	0	17	0	0	0	11	0	17	0	0	11	0	17
12	7	12	1	0	0	12	4	15	0	0	12	0	17
13	12	6	2	1	0	13	0	17	0	0	13	0	17
14	12	8	2	0	0	14	0	17	0	0	14	0	17
15	8	8	2	1	0	15	2	16	0	0	15	0	17
16	19	3	3	0	0	16	0	17	0	0	16	0	17
17	9	11	1	0	0	17	4	15	0	0	17	0	17
18	8	10	2	0	0	18	0	17	0	0	18	0	17
19	1	7	0	1	3	19	4	15	0	0	19	0	17
20	4	5	4	2	0	20	0	17	0	0	20	0	17
21	8	13	0	0	0	21	0	17	0	0	21	0	17
22	4	5	2	1	2	22	0	17	0	0	22	0	17
23	8	5	2	0	2	23	0	17	0	0	23	0	17
24	4	15	0	0	0	24	0	17	0	0	24	0	17
25	6	14	0	0	0	25	0	17	0	0	25	0	17
26	4	5	2	1	2	26	2	16	0	0	26	0	17
27	3	14	1	0	0	27	0	17	0	0	27	0	17
28	4	13	0	1	0	28	2	16	0	0	28	0	17
29	3	13	0	0	1	29	0	17	0	0	29	0	17
30	2	14	0	1	0	30	0	17	0	0	30	0	17
31	0	17	0	0	0	31	2	16	0	0	31	0	17
32	0	17	0	0	0	32	0	17	0	0	32	0	17
33	1	15	1	0	0	33	0	17	0	0	33	0	17
34	1	13	1	1	0	34	2	16	0	0	34	0	17
35	5	9	2	0	1	35	0	17	0	0	35	0	17
36	3	12	1	1	0	36	2	16	0	0	36	0	17

37	0	17	0	0	0	37	0	17	0	0	37	0	17
38	5	10	3	0	0	38	0	17	0	0	38	0	17
39	6	10	1	0	1	39	0	17	0	0	39	0	17
40	5	9	1	2	0	40	0	17	0	0	40	0	17
41	2	12	0	2	0	41	0	17	0	0	41	0	17
42	1	11	1	2	0	42	4	15	0	0	42	0	17
43	2	14	0	1	0	43	0	17	0	0	43	0	17
44	2	14	0	1	0	44	2	16	0	0	44	0	17
45	0	7	0	5	0	45	2	16	0	0	45	0	17
46	3	11	3	0	0	46	0	17	0	0	46	0	17
47	7	12	1	0	0	47	0	17	0	0	47	0	17
48	3	14	1	0	0	48	0	17	0	0	48	0	17
49	3	12	1	1	0	49	0	17	0	0	49	0	17
50	4	12	2	0	0	50	0	17	0	0	50	0	17
51	4	7	0	4	0	51	0	17	0	0	51	0	17
52	4	11	0	2	0	52	0	17	0	0	52	0	17
53	2	14	0	1	0	53	2	16	0	0	53	0	17
54	2	16	0	0	0	54	4	15	0	0	54	0	17
55	1	12	3	0	0	55	0	17	0	0	55	0	17
56	1	13	1	1	0	56	0	17	0	0	56	0	17
57	15	4	1	2	0	57	0	17	0	0	57	0	17
58	0	17	0	0	0	58	0	17	0	0	58	0	17
59	15	5	3	0	0	59	0	17	0	0	59	0	17
60	4	12	2	0	0	60	0	17	0	0	60	0	17
61	8	9	0	2	0	61	0	17	0	0	61	0	17
62	4	12	2	0	0	62	0	17	0	0	62	0	17
63	17	5	1	1	0	63	2	16	0	0	63	0	17
64	11	3	1	1	2	64	0	17	0	0	64	0	17
65	5	10	3	0	0	65	2	16	0	0	65	0	17
66	16	7	0	1	0	66	0	17	0	0	66	0	17
67	6	2	2	2	2	67	2	16	0	0	67	0	17
-	-	-	-	-	-	68	0	17	0	0	68	0	17
-	-	-	-	-	-	69	2	16	0	0	69	0	17
-	-	-	-	-	-	70	0	17	0	0	70	0	17
-	-	-	-	-	-	71	2	16	0	0	71	0	17
-	-	-	-	-	-	72	0	15	0	1	72	0	17
-	-	-	-	-	-	73	0	17	0	0	73	0	17
-	-	-	-	-	-	74	2	16	0	0	74	0	17
-	-	-	-	-	-	75	0	17	0	0	75	0	17
-	-	-	-	-	-	76	2	16	0	0	76	0	17
-	-	-	-	-	-	77	0	15	0	1	77	0	17
-	-	-	-	-	-	78	0	17	0	0	78	0	17
-	-	-	-	-	-	79	2	16	0	0	79	0	17
-	-	-	-	-	-	80	2	16	0	0	80	0	17

-	-	-	-	-	-	81	0	17	0	0	81	0	17
-	-	-	-	-	-	82	0	17	0	0	82	0	17
-	-	-	-	-	-	83	0	17	0	0	83	0	17
-	-	-	-	-	-	84	2	16	0	0	84	0	17
-	-	-	-	-	-	85	0	17	0	0	85	0	17
-	-	-	-	-	-	86	0	17	0	0	86	0	17
-	-	-	-	-	-	87	0	17	0	0	87	0	17
-	-	-	-	-	-	88	0	15	0	1	88	0	17
-	-	-	-	-	-	89	0	17	0	0	89	0	17
-	-	-	-	-	-	90	0	17	0	0	90	0	17
-	-	-	-	-	-	91	0	17	0	0	91	0	17
-	-	-	-	-	-	92	2	16	0	0	92	0	17
-	-	-	-	-	-	93	2	16	0	0	93	0	17
-	-	-	-	-	-	94	0	13	0	2	94	0	17
-	-	-	-	-	-	95	0	17	0	0	95	0	17
-	-	-	-	-	-	96	0	17	0	0	96	0	17
-	-	-	-	-	-	97	0	17	0	0	97	0	17
-	-	-	-	-	-	98	0	17	0	0	98	0	17
-	-	-	-	-	-	99	0	17	0	0	99	0	17
-	-	-	-	-	-	100	0	17	0	0	100	0	17
-	-	-	-	-	-	101	0	17	0	0	101	0	17
-	-	-	-	-	-	102	0	17	0	0	102	0	17
-	-	-	-	-	-	103	3	14	1	0	103	0	17
-	-	-	-	-	-	104	0	17	0	0	104	0	17
-	-	-	-	-	-	105	1	13	1	1	105	0	17
-	-	-	-	-	-	-	-	-	-	-	106	0	17
-	-	-	-	-	-	-	-	-	-	-	107	0	17
-	-	-	-	-	-	-	-	-	-	-	108	0	17
-	-	-	-	-	-	-	-	-	-	-	109	0	17
-	-	-	-	-	-	-	-	-	-	-	110	0	17
-	-	-	-	-	-	-	-	-	-	-	111	0	17
-	-	-	-	-	-	-	-	-	-	-	112	0	17
-	-	-	-	-	-	-	-	-	-	-	113	0	17
-	-	-	-	-	-	-	-	-	-	-	114	0	17
-	-	-	-	-	-	-	-	-	-	-	115	0	17
-	-	-	-	-	-	-	-	-	-	-	116	0	17
-	-	-	-	-	-	-	-	-	-	-	117	0	17
-	-	-	-	-	-	-	-	-	-	-	118	0	17
-	-	-	-	-	-	-	-	-	-	-	119	0	17
-	-	-	-	-	-	-	-	-	-	-	120	0	17
-	-	-	-	-	-	-	-	-	-	-	121	0	17
-	-	-	-	-	-	-	-	-	-	-	122	0	17
-	-	-	-	-	-	-	-	-	-	-	123	0	17
-	-	-	-	-	-	-	-	-	-	-	124	0	17

-	-	-	-	-	-	-	-	-	-	-	125	0	17
-	-	-	-	-	-	-	-	-	-	-	126	0	17
-	-	-	-	-	-	-	-	-	-	-	127	0	17
-	-	-	-	-	-	-	-	-	-	-	128	0	17
-	-	-	-	-	-	-	-	-	-	-	129	0	17
-	-	-	-	-	-	-	-	-	-	-	130	0	17
-	-	-	-	-	-	-	-	-	-	-	131	0	17
-	-	-	-	-	-	-	-	-	-	-	132	0	17
-	-	-	-	-	-	-	-	-	-	-	133	0	17
-	-	-	-	-	-	-	-	-	-	-	134	0	17
-	-	-	-	-	-	-	-	-	-	-	135	0	17
-	-	-	-	-	-	-	-	-	-	-	136	0	17
-	-	-	-	-	-	-	-	-	-	-	137	0	17
-	-	-	-	-	-	-	-	-	-	-	138	0	17
-	-	-	-	-	-	-	-	-	-	-	139	0	17
-	-	-	-	-	-	-	-	-	-	-	140	0	17
-	-	-	-	-	-	-	-	-	-	-	141	0	17
-	-	-	-	-	-	-	-	-	-	-	142	0	17
-	-	-	-	-	-	-	-	-	-	-	143	0	17
-	-	-	-	-	-	-	-	-	-	-	144	0	17
-	-	-	-	-	-	-	-	-	-	-	145	0	17
-	-	-	-	-	-	-	-	-	-	-	146	0	17
-	-	-	-	-	-	-	-	-	-	-	147	0	17
-	-	-	-	-	-	-	-	-	-	-	148	0	17
-	-	-	-	-	-	-	-	-	-	-	149	0	17
-	-	-	-	-	-	-	-	-	-	-	150	0	17
-	-	-	-	-	-	-	-	-	-	-	151	0	17
-	-	-	-	-	-	-	-	-	-	-	152	0	17
-	-	-	-	-	-	-	-	-	-	-	153	0	17
-	-	-	-	-	-	-	-	-	-	-	154	0	17
-	-	-	-	-	-	-	-	-	-	-	155	0	17
-	-	-	-	-	-	-	-	-	-	-	156	0	17
-	-	-	-	-	-	-	-	-	-	-	157	0	17
-	-	-	-	-	-	-	-	-	-	-	158	0	17
-	-	-	-	-	-	-	-	-	-	-	159	0	17
chromosome configurations	6.87	9.99	1.07	0.69	0.24	chromosome configurations	0.70	16.50	0.02	0.06	chromosome configurations	0.01	16.99
Percentage of MMCs with univalent	89.55%					Percentage of MMCs with univalent	29.52%				Percentage of MMCs with univalent	0.63%	

Supplementary Table 2. Pollen in situ germination under different pollination treatments

Treatment	No. of flowers	Total No. of styles	No. of styles			Percentage (%)			No. of styles with pollen tubes going through per flower
			With pollens	With pollen tubes	With pollen tubes go through the style	With pollens	With pollen tubes	With pollen tubes go through the style	
2 days after H30-6 bagging natural pollination	24	116	0	0	0	0.00	0.00	0.00	0.00
3 days after H30-6 bagging natural pollination	23	115	0	0	0	0.00	0.00	0.00	0.00
4 days after H30-6 bagging natural pollination	20	99	0	0	0	0.00	0.00	0.00	0.00
2 days afterH30-6 open pollinate	27	129	28	28	0	21.71	21.71	0.00	0.00
3 days afterH30-6 open pollinate	25	119	49	47	5	41.18	39.50	4.20	0.21
4 days afterH30-6 open pollinate	21	90	42	41	15	46.67	45.56	16.67	0.83
2 days after⊗ H30-6	20	100	1	1	0	1.00	1.00	0.00	0.00
2 days after H30-6 (♀) xH411 (♂)	20	97	86	86	78	88.66	88.66	80.41	4.02
2 days after H30-6 (♀) xB336 (♂)	20	97	44	44	41	45.36	45.36	42.27	2.11

Supplementary Table 3. Embryo sac observation of flowers 1DBO

Lines/ varieties	ovule with normal embryo sac	ovule with abnormal embryo sac	Normal ratio (%)	No. of ovules with normal embryo sac per flower	Each abnormity percentage			
					Tetrad stage	Binucleate embryo sac stage	Without embryo sac	Undetermined abnormity
H30-6	32	110	22.54	2.25	17.27	1.82	68.18	12.73
H411	208	81	71.97	7.20	19.75	11.11	60.49	8.64
B336	118	5	95.93	9.59	80	0	20	0

Supplementary Table 4. Chromosome parameters of the three loquat materials

Lines/varieties	H30-6					H411					B336				
Chromosome number	Relative length				Chromosome type	Relative length				Chromosome type	Relative length				Chromosome type
	Long arm	Short arm	Full length	Arm ratio		Long arm	Short arm	Full length	Arm ratio		Long arm	Short arm	Full length	Arm ratio	
1	4.87±0.27	2.53±0.21	7.39±0.18	1.94±0.10	sm	5.3±0.42	2.34±0.20	7.64±0.31	2.28±0.12	sm	4.95±0.48	2.42±0.40	7.37±0.35	2.08±0.17	sm
2	4.07±0.29	2.87±0.28	6.94±0.11	1.43±0.02	m	4.64±0.46	2.44±0.12	7.09±0.36	1.90±0.09	sm	4.04±0.24	2.90±0.35	6.95±0.08	1.41±0.09	m
3	4.44±0.17	2.25±0.14	6.68±0.14	1.98±0.03	sm	3.92±0.12	2.85±0.07	6.77±0.13	1.38±0.01	m	4.09±0.24	2.51±0.27	6.60±0.14	1.64±0.09	m
4	3.99±0.20	2.45±0.29	6.44±0.13	1.64±0.08	m	4.32±0.08	2.17±0.18	6.49±0.09	1.99±0.06	sm	3.85±0.20	2.63±0.25	6.48±0.06	1.47±0.07	m
5	4.00±0.31	2.27±0.41	6.28±0.10	1.80±0.03	sm	4.07±0.48	2.11±0.47	6.18±0.15	1.98±0.22	sm	3.61±0.27	2.71±0.25	6.32±0.13	1.34±0.08	m
6	3.39±0.09	2.79±0.20	6.18±0.08	1.22±0.03	m	3.97±0.45	2.16±0.49	6.13±0.13	1.90±0.24	sm	3.63±0.32	2.59±0.25	6.22±0.10	1.41±0.08	m
7	3.80±0.21	2.18±0.38	5.98±0.12	1.76±0.03	sm	3.64±0.51	2.44±0.62	6.08±0.07	1.56±0.23	m	3.49±0.34	2.54±0.53	6.03±0.10	1.42±0.15	m
8	3.25±0.25	2.70±0.15	5.95±0.11	1.21±0.04	m	3.46±0.17	2.47±0.34	5.92±0.13	1.41±0.08	m	3.91±0.17	2.06±0.30	5.97±0.13	1.92±0.10	sm
9	3.27±0.26	2.60±0.21	5.87±0.08	1.26±0.07	m	3.84±0.24	2.03±0.22	5.88±0.10	1.90±0.10	sm	3.38±0.30	2.44±0.24	5.82±0.10	1.39±0.08	m

10	3.15±0.29	2.55±0.31	5.70±0.06	1.25±0.07	m	3.27±0.17	2.45±0.21	5.72±0.08	1.34±0.06	m	3.38±0.20	2.36±0.26	5.74±0.10	1.44±0.08	m
11	3.36±0.13	2.25±0.21	5.60±0.08	1.51±0.07	m	3.24±0.13	2.32±0.05	5.56±0.10	1.40±0.02	m	3.30±0.21	2.28±0.19	5.58±0.08	1.46±0.07	m
12	3.03±0.17	2.47±0.12	5.50±0.10	1.24±0.06	m	3.37±0.30	2.06±0.44	5.43±0.07	1.68±0.15	m	3.06±0.25	2.42±0.08	5.49±0.14	1.27±0.05	m
13	3.05±0.15	2.39±0.17	5.44±0.05	1.28±0.06	m	3.05±0.10	2.29±0.18	5.34±0.14	1.34±0.04	m	3.00±0.22	2.38±0.30	5.39±0.10	1.27±0.08	m
14	2.97±0.33	2.37±0.22	5.34±0.17	1.26±0.06	m	3.24±0.39	1.95±0.50	5.19±0.11	1.72±0.19	sm	3.10±0.23	2.18±0.24	5.28±0.06	1.44±0.08	m
15	2.97±0.19	2.15±0.19	5.12±0.21	1.39±0.06	m	2.81±0.22	2.21±0.17	5.02±0.06	1.28±0.06	m	3.10±0.22	2.09±0.34	5.19±0.13	1.50±0.11	m
16	2.81±0.39	2.12±0.20	4.92±0.35	1.33±0.06	m	3.19±0.18	1.76±0.32	4.94±0.14	1.84±0.12	sm	2.69±0.08	2.16±0.24	4.85±0.14	1.25±0.05	m
17	2.58±0.17	2.06±0.09	4.65±0.15	1.26±0.03	m	2.52±0.42	2.11±0.08	4.63±0.30	1.19±0.07	m	2.61±0.14	2.11±0.18	4.72±0.19	1.24±0.03	m

Note: Values are presented as mean ± SE.
